# Ultrasound- and LED-mediated modulation of drying behaviour, surface morphology, phenolic composition, and volatile profiles in goji berries

**DOI:** 10.1016/j.ultsonch.2026.108115

**Published:** 2026-10-13

**Authors:** Muhammad Tayyab Rashid, Nazish Muzaffar, Longquan Fan, Mushtaque Ahmed Jatoi, Abderrahmane Aït-Kaddour, Jasur Safarov, Marwa Ezz El-Din Ibrahim, Rana Muhammad Aadil

**Affiliations:** a School of Health Management, Shanxi Technology and Business University, Taiyuan 030036, China; b College of Food Science and Engineering, Henan University of Technology, Zhengzhou 450001, China; c Department of Botany, Shah Abdul Latif University, Khairpur 65200, Sindh, Pakistan; d Université Clermont Auvergne, INRAE, VetAgro Sup, UMRF, 63370 Lempdes, France; e Department of Food Science and Technology, IPB University, IPB Dramaga Campus, Bogor 16680, West Java, Indonesia; f Tashkent State Technical University Named After Islam Karimov, 100095, 2, Str. University Tashkent, Uzbekistan; g Department of Food and Nutrition Sciences, College of Agricultural and Food Sciences, King Faisal University, Al-Ahsa 31982, Saudi Arabia; h National Institute of Food Science and Technology, University of Agriculture, Faisalabad 38000, Pakistan

**Keywords:** *Lyciumbarbarum*, Ultrasound-assisted drying, Light-emitting diode, Drying kinetics, Phenolic compounds, Volatile profile

## Abstract

Goji berry (*Lycium barbarum L.*) is highly perishable and requires dehydration for preservation, creating challenges for retaining nutritional and functional quality. This study compared blue, yellow, and red light-emitting diode (LED) treatment followed by a common ultrasound pretreatment and far-infrared drying at 70, 80, and 90 °C. Treatment-dependent effects were evaluated for drying behaviour, apparent activation parameters, antioxidant and enzymatic responses, phenolic composition, endpoint surface morphology, and volatile profiles. In the drying profiles at the 90 °C chamber, US-BL90 and US-YL90 reached the final moisture criterion at approximately 300 min, whereas US-Con90 reached it at approximately 540 min. Within the empirical-model comparison, the Hii and Midilli models yielded the strongest goodness-of-fit statistics; the derived apparent activation energies ranged from 23.43 to 31.28 kJ/mol. DPPH radical-scavenging activity was highest in US-Con80 (0.94 mM TE/g), while total phenolic content was highest in US-Con90 (2.42 mg GAE/100 g). US-YL70 showed the highest summed concentration of quantified phenolic compounds among the processed samples. Browning index was highest in US-RL80 (0.61). SEM showed treatment-dependent endpoint external surface features, including surface openings, fissures, compact regions, and differences in wax-like surface features. The applied HS-SPME/GC–MS workflow identified 82 volatile compounds and showed treatment-dependent differences in the measured volatile profiles. Overall, LED treatment within a common ultrasound-assisted far-infrared drying sequence modified dehydration behaviour and multiple quality attributes, but no treatment produced uniformly beneficial responses across all measured outcomes.

## Graphical abstract

**Figure:**
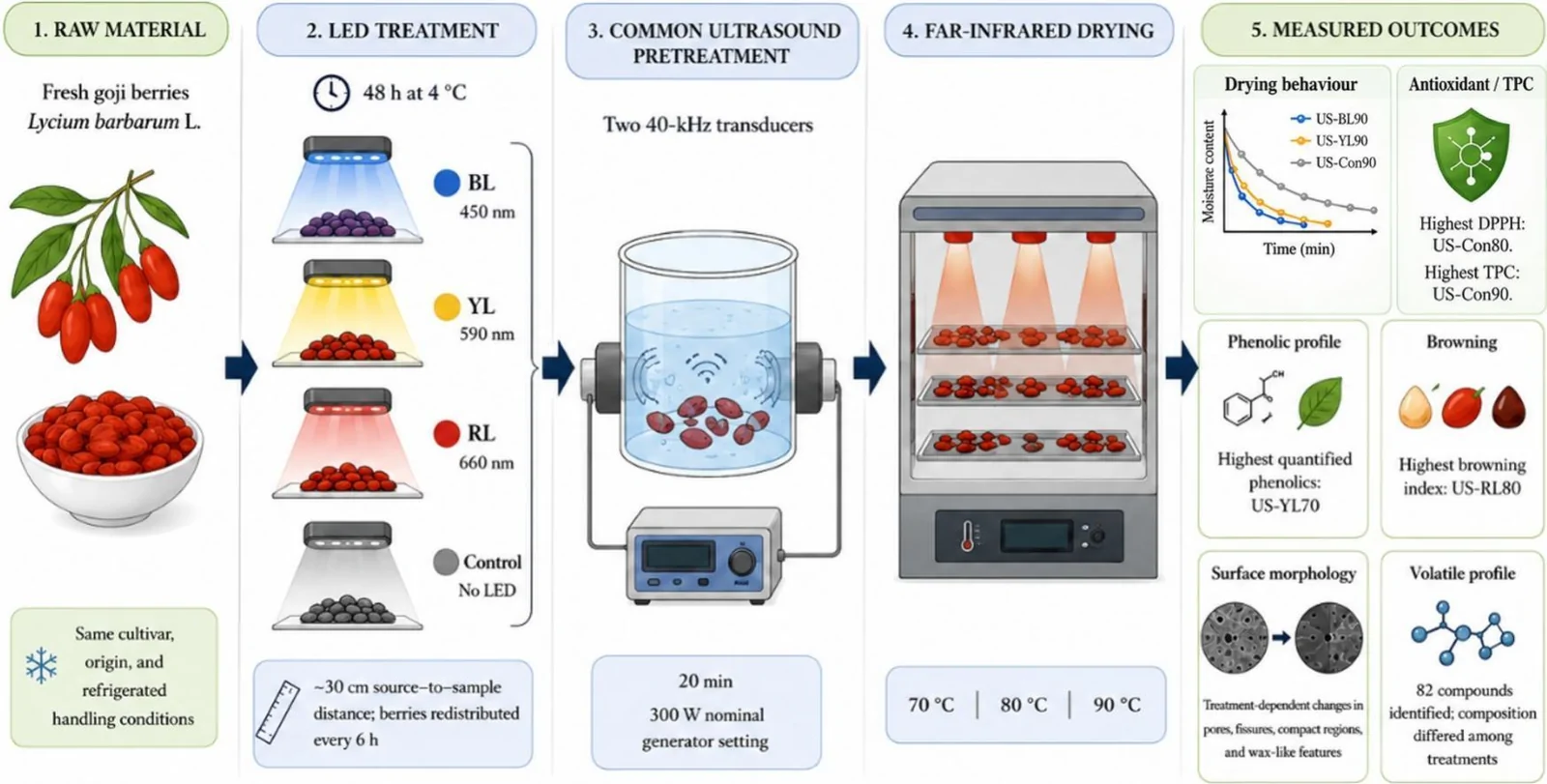

## 1 Introduction

Goji berry is a functional fruit valued for its dense nutritional and phytochemical composition, including polysaccharides, phenolic acids, flavonoids, carotenoids, vitamins, minerals, and antioxidant constituents. These compounds contribute to its reported antioxidant, immunomodulatory, anti-inflammatory, hepatoprotective, neuroprotective, and metabolic regulatory activities, supporting its use in functional foods and nutraceutical products [1]. It's further emphasized that the biological relevance of the goji berry lies in its complex bioactive matrix rather than a single dominant compound [2]. However, fresh goji berries are highly perishable because of their high moisture content, delicate epidermal structure, and active postharvest metabolism, which accelerate microbial spoilage, enzymatic oxidation, softening, browning, and bioactive degradation [3]. Therefore, effective postharvest processing is essential to extend shelf life while preserving functional and sensory quality [4], [5]. Drying is widely used for goji berry preservation because it lowers water activity and limits microbial and biochemical deterioration. Nevertheless, drying can also induce quality losses when heat and exposure time are not properly controlled. Traditional sun drying is inexpensive but often results in non-uniform dehydration, contamination risk, pigment loss, enzymatic browning, and volatile deterioration. Controlled convective drying offers better process control, but prolonged thermal exposure may still cause shrinkage, surface hardening, oxidation of heat-sensitive compounds, and structural collapse [6]. The major challenge in fruit drying is not simply water removal, but efficient dehydration with minimal physical and biochemical damage [7]. This creates the need for drying-intensification strategies that improve internal moisture migration without excessive thermal injury.

Ultrasound-assisted drying has gained attention as a promising approach to overcome internal mass-transfer limitations in fruit tissues. During ultrasound treatment, alternating compression and rarefaction cycles generate cavitation, microstreaming, and sponge-like effects that weaken cell walls, disrupt tissue compactness, and create additional pathways for water migration [8], [9]. These effects can reduce internal diffusion resistance and improve transport of water and soluble constituents within plant matrices [10], [11]. In drying applications, ultrasound pretreatment has been reported to shorten drying time, increase effective moisture diffusivity, and improve selected quality attributes in fruits and vegetables [10], [12]. LED irradiation has also been reported to influence postharvest physiological and biochemical responses in plant tissues, including phenylpropanoid metabolism, antioxidant systems, pigment stability, and stress-related enzyme activity [13], [14]. These reported effects provide a rationale for examining whether prior LED exposure is associated with differences in subsequent drying behaviour, without assigning a specific physiological mechanism to the present treatments. Accordingly, LED treatment before a common ultrasound pretreatment was evaluated as a sequential processing condition, and comparisons were made among the nominal blue-, yellow-, red-LED, and non-irradiated groups within the same ultrasound-treated background. The design does not independently estimate an LED × ultrasound interaction. Ultrasound-assisted far-infrared drying of goji berry has already been reported; notably, Zhang et al. [10] evaluated ultrasonic frequency, power, irradiation height, and drying temperature in relation to drying characteristics, selected quality indices, and microstructure. Other goji studies have examined alternative or hybrid drying approaches with emphasis on structural, antioxidant, or phenolic responses [15], [16], [17]. A focused comparison of the closest goji berry processing studies and their analytical coverage is provided in Supplementary Table S1. Accordingly, novelty is not attributed to ultrasound-assisted far-infrared drying itself, nor to an isolated wavelength effect. The present study instead compares nominal blue-, yellow-, and red-LED treatment conditions within a common subsequent ultrasound and far-infrared drying sequence, across 70, 80, and 90 °C chamber setpoints, while integrating drying behaviour with DPPH radical-scavenging response, total and individual phenolic measurements, relative PPO/POD responses, browning index, endpoint external surface morphology, and volatile-profile characterization.

## 2 Materials & methods

### 2.1 Experimental material

The goji berries used in this study belonged to the Ningxia No. 1 cultivar and were obtained from Ningxia, China. Freshly harvested fruits of this cultivar and geographical origin were transported to the laboratory and maintained at 4 °C before analytical measurements and the subsequent treatment and drying experiments. All experimental treatment groups therefore used goji berries of the same specified cultivar and geographical origin and underwent the same pre-treatment refrigerated holding condition, minimizing cultivar-, provenance-, and storage-related variation when comparing subsequent LED-treatment responses. After this common material-handling step, the berries were allocated to the independently replicated LED-treated and non-irradiated control batches. The initial moisture content of the fresh berries was 78.99% (wet basis).

### 2.2 LED irradiation treatment

LED treatment was performed in corrugated fiberboard containers (45 × 25 × 35 cm) with LED bars mounted above the samples. Three independent batches were prepared for each LED condition and the corresponding control. In each replicate, approximately 1 kg of goji berries was exposed to blue LED (BL; 450 nm), yellow LED (YL; 590 nm), or red LED (RL; 660 nm), or maintained as a non-irradiated control. In the treatment codes, BL, YL, and RL represent blue-, yellow-, and red-LED-treated samples, respectively, while 70, 80, and 90 indicate the subsequent drying temperatures (°C). All groups were maintained at 4 °C for 48 h. LED-treated batches were continuously irradiated with the designated source positioned approximately 30 cm above the berry surface, while controls were kept under the same refrigerated conditions without intentional LED exposure. Berries were redistributed every 6 h to reduce positional variation. Irradiance, photon-flux density, and cumulative photon dose were not independently quantified; accordingly, the groups are referred to as nominal blue-, yellow-, and red-LED treatment conditions rather than dose-normalized wavelength-response treatments. After LED treatment, all samples underwent ultrasound pretreatment.

### 2.3 Ultrasound pretreatment

Goji berries (100 g) were placed in a stainless-steel treatment container (10 × 10 × 10 cm), with deionized water added according to the original experimental protocol. The container was coupled to a two-transducer ultrasound system and ultrasonic generator (Kemeida/Comida Ultrasonic Instrument Co., Ltd., Shenzhen, China) equipped with an infrared temperature sensor. Pretreatment was performed using two 40-kHz transducers operated simultaneously for 20 min at a nominal generator power setting of 300 W. This value represents the generator setting, not calorimetrically determined acoustic power delivered to the sample. Acoustic pressure amplitude, calorimetric acoustic power, and cavitation intensity were not independently quantified. After sonication, berries were drained for 5 min to remove excess surface water, and their moisture content was subsequently determined. Accordingly, ultrasound was treated as a common processing step across the controlled groups rather than as a quantitatively characterized cavitation variable.

### 2.4 Far-infrared drying experiment

Drying was carried out using a far-infrared drying oven (ZKT-210EF, Bai Funuo Machinery Equipment Co., Ltd., Nanjing, China), which was preheated before each drying run to achieve the selected chamber temperature before sample loading. The dryer had external dimensions of 90 × 90 × 175 cm and a working chamber of 60 × 70 × 50 cm. The unit's electrical supply frequency was 50 Hz. For each run, 100 g of ultrasonically pretreated goji berries was uniformly spread on a perforated tray and placed inside the drying chamber. The dryer was operated at chamber setpoints of 70, 80, and 90 °C, and sample mass was recorded at 30 min intervals throughout drying. Drying was terminated when the sample moisture content decreased below 10% (w/w).

### 2.5 Empirical modelling of drying behaviour

The experimental drying data were fitted to 13 empirical thin-layer models listed in Supplementary Table S2 to compare their ability to reproduce the moisture-ratio trajectories under different treatment conditions. These models were used as descriptive curve-fitting tools rather than direct physical representations of heat- and mass-transfer mechanisms. The moisture ratio (MR) and drying rate (DR) during drying were calculated using Eqs. (1), (2), respectively.

(1) $$\text{MR}\;=\;\left(M_{t}-M_{e}\right)\;/\;\left(M_{0}-M_{e}\right)$$

In this equation, *t* denotes drying time (s), whereas M_0_, M_e_, and M_t_ represent the initial moisture content, equilibrium moisture content, and moisture content at time t (g/g), respectively.

The drying rate (DR) was calculated from the change in moisture content between two consecutive mass measurements according to Eq. (2):

(2) $$\text{DR}\;=\;\left(M_{t}-M_{t+\Delta t}\right)\;/\;\Delta \text{t}$$

where M_t_ and M_t_+Δt are the moisture contents on a dry-matter basis (kg water kg⁻^1^ dry matter) at times t and t + Δt, respectively. Because sample mass was recorded every 30 min, Δt was expressed in hours (0.5 h for each interval) when calculating DR; accordingly, DR is reported as kg water kg⁻^1^ dry matter h⁻^1^ and represents an interval-averaged rather than an instantaneous rate.

### 2.6 Apparent activation energy of the drying kinetics

The apparent activation energy (Ea) was estimated from the Arrhenius relationship between the drying constant obtained from the selected empirical model and absolute temperature. Because the fitted drying constant is an empirical kinetic parameter, the resulting Ea is interpreted as an apparent activation parameter describing the temperature sensitivity of the fitted drying process rather than as a direct measurement of effective moisture diffusivity or equilibrium water-binding energy:

(3) $$k=k_{0}exp\left(-\frac{E_{a}}{R_{g}T}\right)$$

where *k* k is the drying constant obtained from the selected drying model, *k* 0 k 0 ​ is the pre-exponential factor, *E a* E a ​ is the activation energy (kJ/mol), *R g* ​ is the universal gas constant (8.31451 J/mol·K), and *T* T is the absolute temperature (K).

### 2.7 Apparent activation thermodynamic parameters

Apparent activation enthalpy (ΔH‡), activation entropy (ΔS‡), and activation Gibbs free energy (ΔG‡) were derived from the temperature-dependent kinetic parameters using Eqs. (4), (5), (6). All temperature-dependent calculations used absolute temperature in Kelvin. These quantities are treated as activation-state descriptors associated with the fitted drying kinetics and are interpreted separately from equilibrium sorption thermodynamics and process-level exergy performance.

(4) $$\Delta H=E_{a}-RT$$

(5) $$\Delta S=R\left[ln\left(D_{0}\right)-ln\left(\frac{k_{b}}{h_{p}}\right)-ln\left(T\right)\right]$$

(6) ΔG=ΔH-TΔS

where Δ*H* ΔH is the enthalpy change (kJ/mol), Δ*S* ΔS is the entropy change (kJ/mol·K), Δ*G* ΔG is the Gibbs free energy (kJ/mol), *k b* k b ​ is Boltzmann's constant, and ℎ*p* h p ​ is Planck's constant.

### 2.8 Calculated dryer-stage energy requirement

The present energy was calculated using far-infrared drying stage and included the heating-unit and blower contributions defined in Eqs. (7), (8). The heating-unit contribution was estimated from air flow rate, air density, specific heat capacity, temperature difference, and drying time using Eq. (7). Accordingly, the resulting values are used as comparative dryer-stage energy estimates within this calculation boundary.

(7) $$E_{e}=A*v*rho_{a}*C_{a}*\Delta T*t$$

The blower contribution was calculated using Eq. (8):

(8) $$E_{b}=\frac{v^{3}}{16600}$$

### 2.9 Model fitting and statistical evaluation

Model performance was summarized using R^2^, RMSE, RSS, and reduced χ^2^ by comparing experimental and predicted moisture ratios. These statistics were used to compare empirical fit quality, with goodness of fit interpreted as predictive adequacy rather than evidence of a specific transport mechanism.

### 2.10 Quality attributes

#### DPPH radical-scavenging activity and total phenolic content

Extracts were prepared before antioxidant and phenolic analyses. Briefly, 2.5 g of the dried goji berry sample was mixed with 5 mL of 68% (v/v) aqueous ethanol and sonicated at 40 kHz and 60 °C for 60 min. The mixture was centrifuged at 4000 rpm for 10 min, and the supernatant was concentrated using a rotary evaporator at 60 °C. The obtained extracts were stored in brown bottles at − 25 °C until analysis. Total phenolic content (TPC) was determined and expressed as mg GAE/100 g dry matter. DPPH radical-scavenging activity was evaluated using the DPPH assay and expressed as mM TE/g dry matter [[18], [19]]. All treatment comparisons used the same extraction and analytical procedure.

#### 2.11.1 Relative PPO/POD responses and browning index

Crude enzyme extracts were prepared according to the ultrasound-assisted infrared drying procedure [19]. Briefly, 5 g of powdered dried goji berry was mixed with 25 mL extraction solution containing 0.2 M phosphate buffer (pH 6.5), 1% (v/v) Triton X-100, and 4% polyvinylpolypyrrolidone, mixed for 3 min, held at 4 °C for 4 h, and centrifuged at 10,000 rpm for 10 min at 4 °C. The supernatant was filtered through a 0.45 µm membrane. The PPO assay response was determined by mixing 0.5 mL extract with 0.2 mL 0.1 M catechol and 1.5 mL 0.1 M sodium phosphate buffer (pH 7.0), with absorbance monitored at 420 nm for 5 min; an enzyme-free mixture served as the blank [19]. The POD assay response was measured using 0.15 mL 4% (v/v) guaiacol, 0.15 mL 1% (v/v) H2O2, 2.66 mL 0.1 M phosphate buffer (pH 7.0), and 40 µL extract, with absorbance recorded at 470 nm and an enzyme-free blank [[19], [20]]. PPO and POD results were expressed as relative-response percentages (%) according to Eq. (9) and interpreted comparatively across treatment conditions:

(9) $$Relativeactivity\left(\%\right)=\left(\frac{\mathrm{Currentenzymeactivity}}{\mathrm{Initialenzymeactivity}}\right)\times 100$$

The browning index (BI) was determined according to Sarpong et al. [21], with absorbance recorded at 420 nm. BI was calculated using the following equation:

(10) $$\mathrm{BI}=\frac{100\left(x-0.31\right)}{0.17}$$

### 2.12 HPLC analysis of phenolic profile

Phenolic compounds were analyzed using a Waters E2695 HPLC system (Waters Corporation, USA) equipped with a UV–Vis photodiode-array detector, following the chromatographic procedure reported in Ref. [22]. The reported compound concentrations represent endpoint chromatographic measurements obtained under the same analytical procedure for all samples.

### 2.13 Analysis of volatile compounds by HS-SPME/GC–MS

Volatile compounds were analyzed using headspace solid-phase microextraction coupled with gas chromatography-mass spectrometry (HS-SPME/GC–MS). Briefly, 2.5 g of the sample was transferred into a 20 mL sealed glass vial containing 5.0 g sodium sulfate and 100 μL of 3-octanol (50 ng/mL) as the internal standard. Volatile compounds were extracted using a 50 μm DVB/CAR/PDMS SPME fiber (Supelco, PA, USA) at 60 °C for 30 min. The fiber was subsequently introduced into the GC injection port and thermally desorbed at 250 °C for 5 min using an automated sampler. Chromatographic separation, mass-spectrometric acquisition, compound assignment, and data processing followed the procedure described by Liu et al. [23]. The present analysis was used to compare treatment-dependent volatile profiles across samples.

### 2.14 Scanning electron microscopy (SEM) analysis

Surface morphology of dried goji berry samples was examined using SEM (JEOL JSM-5800LV, Japan). Samples were mounted on SEM stubs, sputter-coated with gold using a sputter coater (SCD050, BALZERS), and observed at selected magnifications to compare endpoint surface-morphological features among treatments.

### 2.15 Statistical analysis

Each treatment condition was performed using three independent processing replicates. For each replicate, a separately prepared batch underwent the specified LED treatment, ultrasound pretreatment, and subsequent far-infrared drying procedure; each replicate was treated as the experimental unit for statistical analysis. Statistical comparisons among the processed treatment groups were performed using two-way ANOVA with LED treatment (US-Con, US-BL, US-YL, and US-RL) and drying temperature (70, 80, and 90 °C) as fixed factors, followed by Tukey's multiple-comparison test at P < 0.05. Multivariate analyses, including PCA, Pearson correlation, and Hierarchical Cluster Analysis (HCA), were performed using OriginPro 2024 to evaluate relationships among measured phenolic compounds, DPPH/TPC, relative PPO/POD responses, browning index, and treatment conditions.

## 3 Results & discussion

### 3.1 Moisture ratio

The moisture ratio (MR) of goji berries decreased progressively (Fig. 1) with drying time under all treatments. The profiles were consistent with a predominantly falling-rate drying pattern, while the present data were used to describe the observed dehydration response rather than to identify a unique internal transport mechanism. Increasing the chamber setpoint from 70 to 90 °C was associated with faster MR reduction and shorter observed drying times across the treatment groups. In the ultrasound control groups, the final drying time decreased from approximately 720 min (US-Con70) to 660 min (US-Con80) and 540 min (US-Con90), demonstrating a strong temperature dependence of the observed dehydration behaviour. Similar temperature-dependent drying behaviour has been widely reported during convective dehydration of berry fruits and other high-moisture plant tissues [[10], [24]]. Because the same ultrasound pretreatment was applied to all controlled drying groups, between-group differences are interpreted within a common ultrasound-treated background and are not used to estimate an independent ultrasound contribution. Among the reported profiles, US-YL90 reached the final moisture criterion at approximately 300 min, followed by US-YL80 (∼420 min) and US-YL70 (∼540 min). US-BL90 likewise reached the criterion at approximately 300 min, while US-BL80 required approximately 420 min. At 90 °C, these observed endpoint times can be compared directly with approximately 540 min for US-Con90. In contrast, the red-light-treated samples exhibited comparatively slower MR reduction and required longer drying times, especially at lower temperatures. These differences document treatment-dependent drying responses among the nominal LED conditions but do not establish the physiological or transport mechanism responsible for them. The MR trends were considered alongside endpoint SEM features such as openings, fissures, and compact surface regions; these observations provide morphological context but do not quantify internal moisture transport. Smoother and more compact endpoint surfaces were observed in some high-temperature treatments (US-BL90, US-YL90, and US-RL90) without being used as evidence of case hardening or later-stage diffusion resistance.

**Fig. 1:**
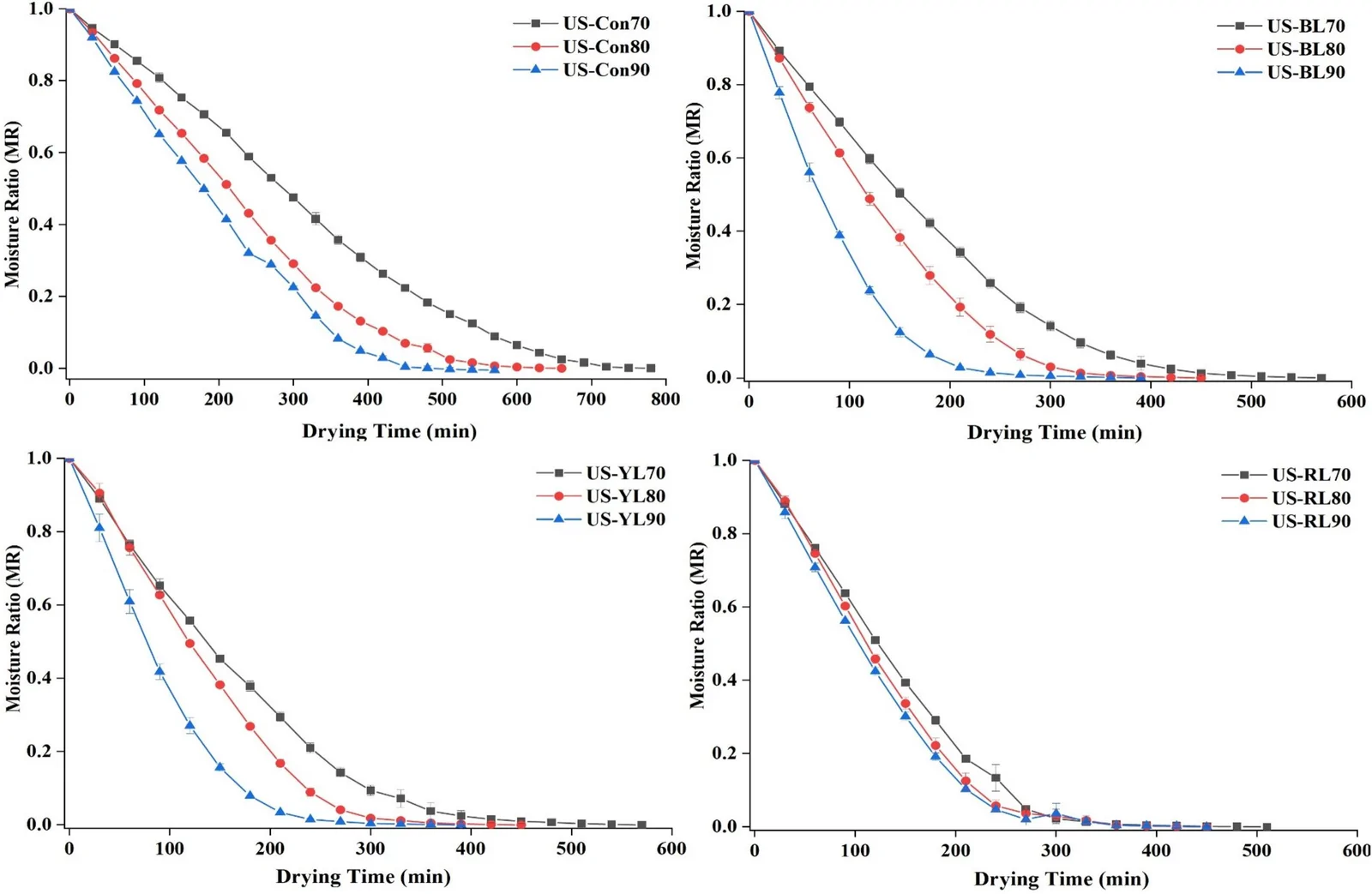
Moisture ratio (MR) curves of goji berries subjected to LED treatment, common ultrasound pretreatment, and far-infrared drying at 70, 80, and 90 °C.

### 3.2 Drying rate

The calculated drying-rate (DR) profiles showed an initial increase followed by a progressive decline under all treatment conditions (Fig. 2). Because DR was determined from consecutive moisture measurements, the plotted values represent interval-averaged moisture-removal rates over the corresponding sampling periods. The subsequent decline in DR is consistent with progressively slower moisture removal as drying proceeded. Similar drying behaviour has been widely reported in berry fruits and other biological materials [[10], [25]]. Increasing the far-infrared drying chamber setpoint from 70 to 90 °C generally increased the calculated DR and shortened the time required to reach the final moisture level. In the ultrasound-treated control series, the reported peak DR increased from approximately 2.1 kg water kg⁻^1^ dry matter h⁻^1^ in US-Con70 to approximately 3.3 kg water kg⁻^1^ dry matter h⁻^1^ in US-Con90, indicating faster moisture removal at the higher chamber setpoint. These DR values describe the observed rate of moisture loss and are not interpreted as direct measurements of effective moisture diffusivity. A comparable temperature-dependent enhancement in DR has been reported during the drying of wolfberries and other fruit tissues subjected to convective dehydration [10]. All US-Con and LED-treated groups received the same ultrasound step; consequently, the DR comparisons describe differences among treatment conditions and drying setpoints rather than an independently estimated ultrasound effect. Among the evaluated profiles, US-BL90 and US-YL90 showed the highest plotted DR values, exceeding 6.0 kg water kg⁻^1^ dry matter h⁻^1^, followed by US-RL80 and US-RL90. Within-temperature comparisons were used when describing differences among the LED treatment conditions. The treatment-dependent DR differences are reported descriptively and are not assigned to a specific LED-mediated physiological or moisture-transport mechanism. After the maximum was reached, DR declined progressively in all treatments as drying proceeded. This interval-averaged response documents the slowing of moisture removal but does not, by itself, discriminate among possible contributions from internal transport, external resistance, tissue shrinkage, or changes in water-binding state. The sharper late-stage decline observed particularly in US-BL90 and US-YL90 occurred alongside enlarged openings and fractured endpoint surface features seen by SEM; this concurrence is treated as an association rather than as evidence that the visible surface features caused the DR trajectory. Similar reductions in DR during advanced dehydration stages have been reported in dried fruit tissues [[25], [26]].

**Fig. 2:**
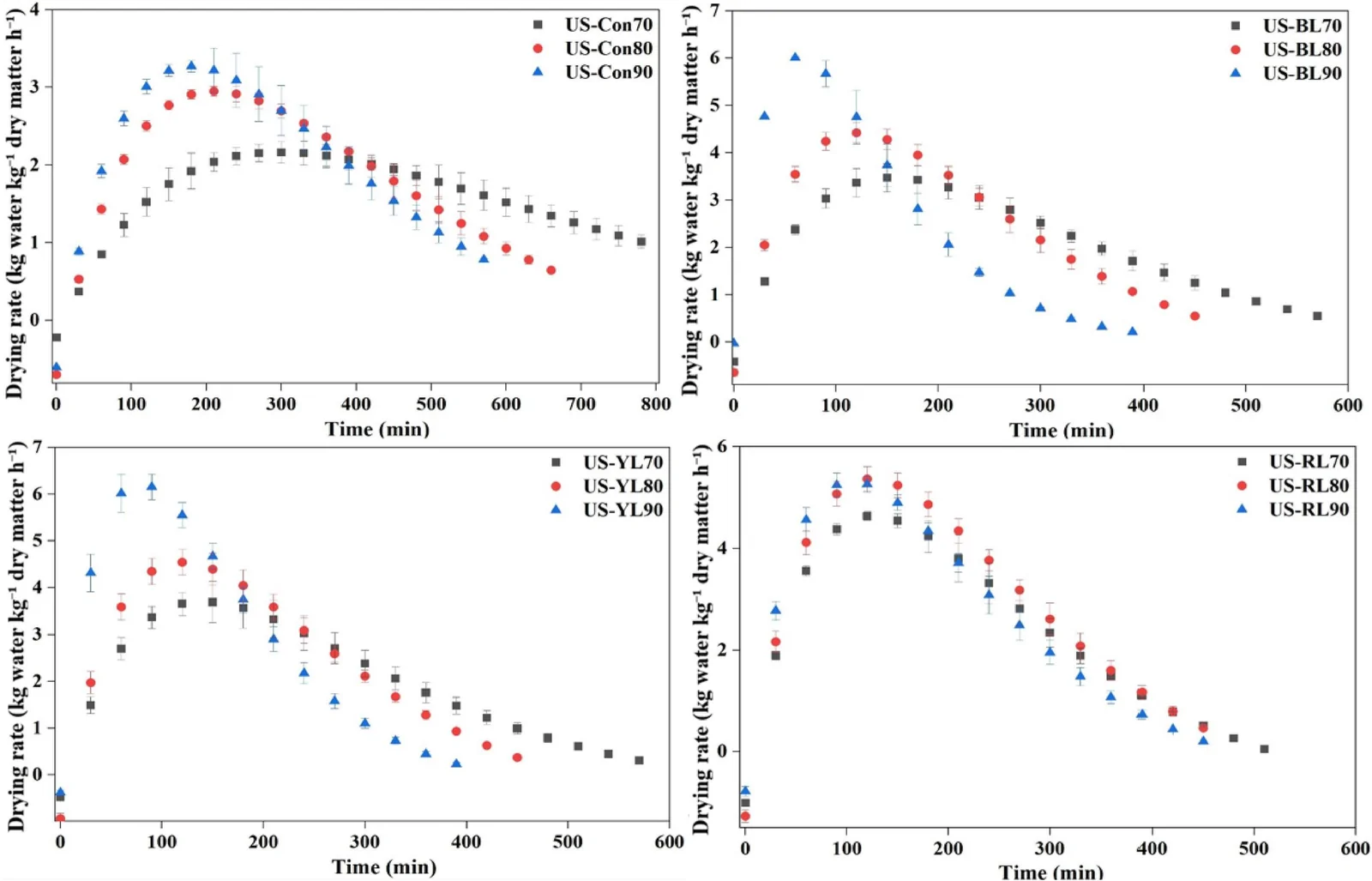
Interval-averaged drying-rate (DR; kg water kg⁻^1^ dry matter h⁻^1^) profiles of goji berries subjected to LED treatment, ultrasound pretreatment, and far-infrared drying at 70, 80, and 90 °C.

### 3.3 Mathematical modelling

The suitability of thin-layer drying models for describing the drying behaviour of goji berries was evaluated based on statistical indicators. Within the reported goodness-of-fit statistics, the Midilli and Hii formulations showed the closest agreement with the experimental moisture-ratio data (Table 1); the remaining formulations are presented in Supplementary Table S3. For the Midilli formulation, the reported high coefficient of determination and low error indices indicate close empirical agreement with the observed moisture-ratio trajectory (Fig. 3), while its combined exponential and linear terms provide flexibility for representing changes in curve shape during drying [[27], [28]]. Such flexibility is useful for heterogeneous biological materials in which the observed drying curve changes continuously as moisture content and tissue structure evolve [29]; nevertheless, the fitted terms are retained as descriptive parameters and are not assigned uniquely to diffusion, shrinkage, or any other physical transport mechanism. The Hii formulation showed similarly close agreement in the reported statistical indicators; its multi-term exponential form provides empirical flexibility for representing curvature across different portions of the drying trajectory [30], without assigning the individual fitted terms to distinct moisture-migration stages. The empirical fits are therefore interpreted only as descriptions of the observed curve shape and do not resolve the physical origin of the treatment-dependent differences. The remaining formulations showed less favorable reported goodness-of-fit statistics for the observed moisture-ratio trajectories. Because the analysis is empirical, differences in goodness of fit are not used to identify the controlling heat- or mass-transfer mechanism [[31], [32]].

**Table 1:** Averages of selected models fitted to the ultrasound-treated Goji berries.

**Sample Codes**	**Coefficients**	**R^2^**	**RSS**	**χ^2^**	**RMSE**
	**1, Hii Model**				
US-Con70 US-Con80 US-Con90 US-BL70 US-BL80 US-BL90 US-YL70 US-YL80 US-YL90 US-RL70 US-RL80 US-RL90	a. 0.692, k1. 2.975 × 10^-05^, n. 1.774, b. 0.268, k2. 2.883 × 10^-05^ a. 0.651, k1. 6.705 × 10^-05^, n. 1.722, b. 0.307, k2. 6.688 × 10^-05^ a. 0.643, k1. 0.00, n. 1.672, b. 0.313, k2. 0.000 0a.0.639, k1. 0.00, n. 1.529, b. 0.325, k2. 0.000 0a.0.96, k1. 1.483, n. 1.738, b. 0.904, k2. 0.000 0a.0.614, k1. 0.003, n. 1.282, b. 0.380, k2. 0.003 0a.0.618, k1. 0.000, n. 1.536, b. 0.344, k2. 0.000 0a.0.325, k1. 0.000, n. 1.668, b. 0.654, k2. 0.000 0a.0.619, k1. 0.001, n. 1.457, b. 0.371, k2. 0.001 0a.0.617, k1. 0.000, n. 1.644, b. 0.352, k2. 0.000 0a.0.067, k1. 1.467, n. 1.797, b. 0.933, k2. 0.000 0a.0.621, k1. 0.001, n. 1.557, b. 0.357, k2. 0.001	0.998 0.998 0.994 0.998 0.999 0.991 0.994 0.998 0.999 0.997 0.999 0.998	0.01 0.01 0.01 0.01 0.00 0.01 0.01 0.00 0.00 0.01 0.00 0.00	0.0007 0.0007 0.0022 0.0005 0.0003 0.0011 0.0013 0.0004 0.0003 0.0005 0.0001 0.0005	0.0170 0.0168 0.0258 0.0169 0.0116 0.0299 0.0246 0.0166 0.0096 0.0176 0.0096 0.0142
	**2, Midilli Model**				
US-Con70 US-Con80 US-Con90 US-BL70 US-BL80 US-BL90 US-YL70 US-YL80 US-YL90 US-RL70 US-RL80 US-RL90	a. 0.970, k. 5.739 × 10^-05^, n. 1.647, b. 6.551 × 10^-05^ a. 0.968, k. 0.000, n. 1.616, b. 4.556 × 10^-05^ a. 0.973, k. 0.000, n. 1.501, b. 9.557 × 10^-05^ a. 0.974, k. 0.000, n. 1.447, b. 4.300 × 10^-05^ a. 0.979, k. 0.001, n. 1.466, b. 5.193 × 10^-05^ a. 0.996, k. 0.003, n. 1.249, b. 3.523 × 10^-05^ a. 0.968, k. 0.000, n. 1.477, b. 3.003 × 10^-05^ a. 0.983, k. 0.000, n. 1.624, b. 3.139 × 10^-05^ a. 0.991, k. 0.001, n. 1.444, b. 1.328 × 10^-05^ a. 0.972, k. 0.000, n. 1.609, b. 2.202 × 10^-05^ a. 0.983, k. 0.000, n. 1.629, b. 1.319 × 10^-05^ a. 0.980, k. 0.001, n. 1.539, b. 1.281 × 10^-05^	0.999 0.998 0.997 0.998 0.998 0.992 0.995 0.998 0.998 0.998 0.999 0.998	0.00 0.00 0.01 0.00 0.00 0.01 0.01 0.00 0.00 0.00 0.00 0.00	0.0003 0.0004 0.0013 0.0003 0.0004 0.0011 0.0012 0.0004 0.0002 0.0004 0.0002 0.0005	0.0114 0.0131 0.0198 0.0138 0.0137 0.0298 0.0234 0.0151 0.0088 0.0162 0.0125 0.0141

**Fig. 3:**
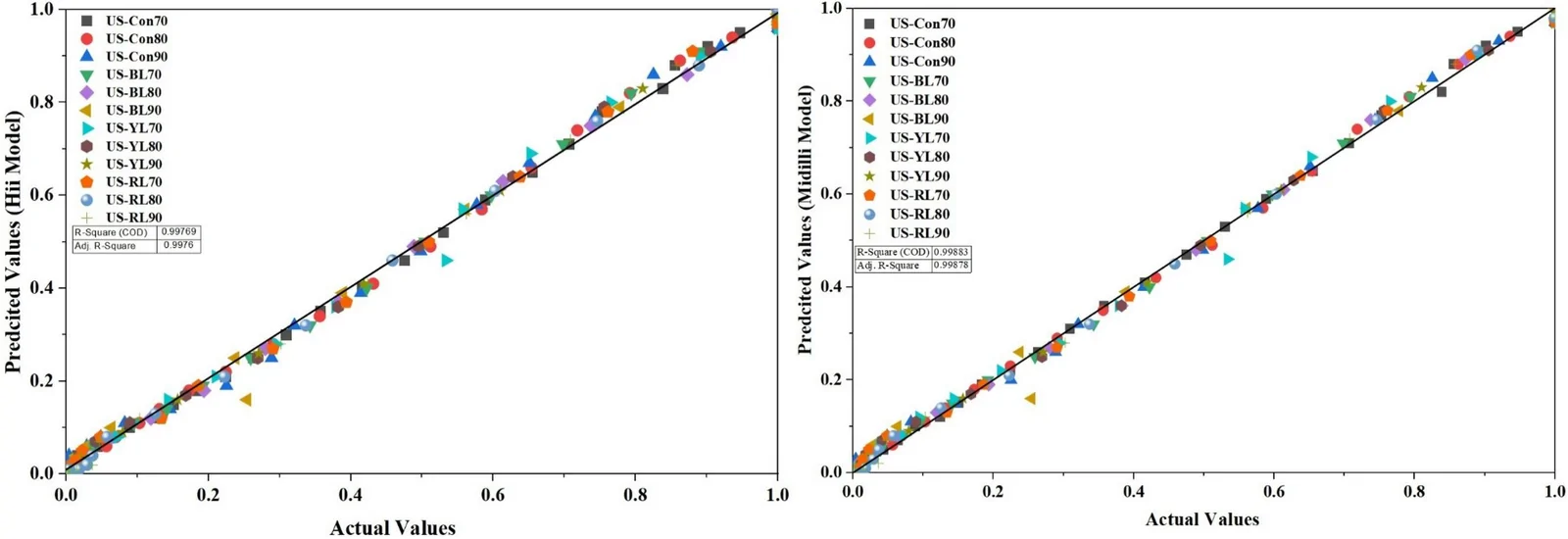
Experimental moisture-ratio data and empirical predictions obtained using the Hii and Midilli models for LED-treated, ultrasound-treated goji berries during far-infrared drying.

### 3.4 Activation energy

The apparent activation-energy (Ea) values in Table 2 describe the temperature sensitivity of the drying constants obtained from the selected kinetic model under the different treatment conditions. Differences among treatments therefore indicate differences in the temperature dependence of the fitted drying behaviour. Still, they should not be interpreted directly as quantitative changes in effective moisture diffusivity or internal mass-transfer resistance. The control samples showed an intermediate Ea (26.48 kJ/mol), whereas the blue and yellow LED treatments yielded higher Ea (∼31 kJ/mol). In contrast, the red-LED treatment exhibited the lowest apparent Ea (23.43 kJ/mol), indicating a lower temperature sensitivity of the fitted kinetic parameter than that observed for the blue- and yellow-LED treatments. In the present analysis, Ea was derived from an empirical drying constant and is therefore used primarily as a comparative kinetic descriptor. Direct conclusions about water–solid binding strength, effective diffusivity, or specific internal transport resistance require independently determined transport or sorption parameters and cannot be inferred from Ea alone [33]. The lower apparent Ea observed in the red-LED treatment is interpreted as a treatment-specific difference in the temperature sensitivity of the fitted drying kinetics. Because ultrasound was common to all controlled groups, no independent ultrasound contribution is assigned to these apparent Ea differences. Accordingly, the Ea difference is discussed as a kinetic response rather than as a direct quantitative measure of cavitation intensity or effective moisture diffusivity. In contrast, the higher apparent Ea values in the blue- and yellow-LED treatments indicate greater temperature sensitivity of the fitted drying constant relative to the red-LED treatment [34]. The apparent Ea values obtained in this study fall within the range reported for drying kinetics of comparable food materials [35].

**Table 2:** Apparent activation energy, activation thermodynamic parameters, and calculated dryer-stage energy estimates of LED-treated, ultrasound-pretreated goji berries subjected to far-infrared drying.

**Sample codes**	**Temperature** **(^o^C)**	**Activation Energy (kJ/mol)**	**R^2^**	ΔH‡ (kJ/mol)	ΔG‡ (kJ/mol)	ΔS‡ (J/mol·K)	**Calculated Energy Estimate** **(kWh/kg)**
US-Con US-Con US-Con	70 80 90	26.48	0.9989	23.63 23.5423.46	193.21 198.14203.23	−494.19 −494.41 −495.03	45.76 33.44 33.44
US-BL US-BL US-BL	70 80 90	31.24	0.9167	28.39 28.3028.22	191.23 196.51200.85	−474.56 −476.29 −475.37	29.92 38.72 26.40
US-YL US-YL US-YL	70 80 90	31.28	0.9979	28.43 28.3428.26	191.24 196.12200.86	−474.46 −475.08 −475.28	26.40 26.40 33.44
US-RL US-RL US-RL	70 80 90	23.43	0.9977	20.58 20.4920.41	190.80 195.77200.86	−496.05 −496.32 −496.89	22.88 22.88 26.40

Con = Control; US-BL/YL/RL = Ultrasound blue, yellow, and red LED goji berry samples.

### 3.5 Calculated dryer-stage energy requirement

The dryer-stage energy estimates in Table 2 varied across drying temperatures and treatment conditions. In the ultrasound-control series, the calculated value decreased from 45.76 kWh/kg at 70 °C to 33.44 kWh/kg at both 80 and 90 °C. The corresponding values for US-BL were 29.92, 38.72, and 26.40 kWh/kg at 70, 80, and 90 °C, respectively, whereas US-YL showed 26.40, 26.40, and 33.44 kWh/kg. The US-RL series showed values of 22.88, 22.88, and 26.40 kWh/kg across the same temperature sequence. Within the present calculation, these differences reflect the combined influence of drying duration and the heating-unit and blower terms used for the far-infrared drying stage. Drying duration is an important component of stage-level energy demand because the operating period directly affects cumulative energy requirements [[36], [37]]. The comparison is therefore confined to the dryer-stage estimates reported in Table 2 and is not used to assign differences in effective moisture diffusivity, internal transport resistance, or overall process energy efficiency to the LED treatment conditions. This treatment-specific pattern provides a bounded comparison of the calculated drying-stage requirement under the operating conditions evaluated in this study.

### 3.6 Apparent activation thermodynamic parameters

The apparent activation thermodynamic parameters presented in Table 2 were derived from the temperature dependence of the fitted drying kinetics and are therefore interpreted within an activation-state framework. The calculated ΔH‡ values showed only small temperature-dependent changes within each treatment, as expected from their relationship with Ea and absolute temperature. Differences among treatments reflect differences in the apparent kinetic barrier represented by the selected drying model and are not interpreted as direct measurements of equilibrium water-binding strength. Positive ΔG‡ values were obtained for all evaluated conditions. In the present context, these values describe the free-energy barrier associated with the apparent activated state of the fitted kinetic process and are conceptually distinct from the overall Gibbs free-energy change of moisture removal. The calculated ΔS‡ values were negative under the investigated conditions and are treated as activation-state parameters associated with the kinetic formulation rather than as direct measures of macroscopic structural ordering in the drying berry matrix. Accordingly, interpretation of ΔH‡, ΔS‡, and ΔG‡ is confined to comparative activation-state behaviour of the fitted kinetics and is kept distinct from equilibrium sorption quantities and process-level exergy performance.

### 3.7 DPPH radical-scavenging activity and total phenolic content

The statistical response differed between DPPH radical-scavenging activity and total phenolic content (TPC). Two-way ANOVA revealed significant effects of temperature (T), LED treatment (L), and their interaction (T × L) on DPPH activity. In contrast, TPC was not significantly affected by these factors (Table 3), showing that the two analytical endpoints did not follow the same statistical pattern. The fresh and sun-dried samples showed identical DPPH values (0.62 mM TE/g). In contrast, TPC increased from 2.01 to 2.23 mg GAE/100 g dry matter after sun drying, while DPPH remained unchanged. The unchanged DPPH response despite the increase in measurable TPC after sun drying illustrates that total Folin-reactive phenolics and DPPH radical-scavenging capacity did not vary in parallel under the applied analytical conditions. In the ultrasound control series, DPPH increased from US-Con70 (0.81 mM TE/g) to the highest value at US-Con80 (0.94 mM TE/g), before declining at US-Con90 (0.73 mM TE/g), while TPC increased continuously from US-Con70 (2.23 mg GAE/100 g) to US-Con80 (2.26 mg GAE/100 g) and US-Con90 (2.42 mg GAE/100 g). This pattern indicates that the ultrasound-treated control conditions produced temperature-dependent differences in measurable TPC and DPPH response [38]. The observed increase in TPC is reported as a change in measurable Folin-reactive phenolic content and is not used to infer a specific release mechanism. At 90 °C, the lower DPPH value despite the higher TPC shows that the two analytical endpoints did not change in parallel under the applied conditions [39]. Across the processed groups, DPPH differed significantly with LED treatment, drying temperature, and their interaction (P < 0.05), whereas TPC showed no significant main or interaction effects in the two-way ANOVA (Table 3). The LED-treated samples showed DPPH values of 0.64–0.67 mM TE/g, while TPC values remained within 2.25–2.42 mg GAE/100 g dry matter. Across the LED-treated groups, DPPH values were lower than in the corresponding ultrasound-treated controls despite comparatively stable TPC values [40], further showing that the two measured endpoints responded differently. Accordingly, these treatment comparisons are discussed in terms of the measured TPC and DPPH endpoints rather than inferred chemical transformations [41]. Among the reported values, US-Con80 had the highest DPPH response, whereas US-Con90 had the highest TPC within the ultrasound-control series [42].Table 4a.Table 4b.

**Table 3:** DPPH, TPC, relative PPO/POD responses, and browning index of goji berries across the evaluated processing conditions.

**Samples**	**DPPH (mM TE/g)**	**TPC (mg GAE/100 g)**	**Relative PPO (%)**	**Relative POD (%)**	**BI**
Fresh	0.62d	2.01b	0.00 g	0.00 g	0.03 g
Sun-dried	0.62d	2.23ab	142.37f	9.98 fg	0.21de
US-Con70	0.81b	2.23ab	166.57f	215.26e	0.11f
US-Con80	0.94a	2.26ab	330.81d	748.92a	0.15f
US-Con90	0.73c	2.42a	196.50f	303.33d	0.18e
US-BL70	0.65d	2.33a	253.26e	391.78c	0.17e
US-BL80	0.65d	2.27ab	267.35e	781.80a	0.25d
US-BL90	0.67 cd	2.32a	279.01de	407.05c	0.35c
US-YL70	0.64d	2.33a	314.58d	593.74b	0.20e
US-YL80	0.64d	2.33a	271.43de	717.61a	0.35c
US-YL90	0.67 cd	2.42a	489.31c	532.09b	0.47b
US-RL70	0.67 cd	2.26ab	238.48e	91.00f	0.23d
US-RL80	0.64d	2.25ab	700.10a	181.60e	0.61a
US-RL90	0.64d	2.25ab	562.78b	222.50de	0.48b
**ANOVA**					
Temperature	0.0035**	0.1182 ns	< 0.0001***	< 0.0001***	< 0.0001***
LED	< 0.0001***	0.1397 ns	< 0.0001***	< 0.0001***	< 0.0001***
Temp × LED	< 0.0001***	0.5184 ns	< 0.0001***	< 0.0001***	< 0.0001***

Different letters within a column indicate significant differences among samples (Tukey test, p < 0.05).

Two-way ANOVA was performed for processed samples only (US-Con, US-BL, US-YL, US-RL at 70, 80, and 90C). T, temperature; L, LED; T × L, interaction.

**Table 4a:** (Part A). Effects of LED irradiation and ultrasound-assisted far-infrared drying on the phenolic profile of goji berries.

**Compounds**	**RT**	**Concentration (µg g⁻^1^ dm)**								**ANOVA**		
	**(min)**	**Fresh**	**Sun-dried**	**US-Con70**	**US-Con80**	**US-Con90**	**US-BL70**	**US-BL80**	**US-BL90**	**T**	**L**	**T × L**
**Phenolic acids**												
GA	0.95	0.0194f	0.1231c	0.1279c	0.0803e	0.1092c	0.0815d	0.1359b	0.1442b	<0.0001***	<0.0001***	<0.0001***
4-HBA	3.36	0.7158 g	4.9798a	2.1306f	3.2306c	2.1668f	2.8113d	2.5836e	3.3116c	<0.0001***	<0.0001***	<0.0001***
VA	4.13	0.2171e	0.6495b	0.4644d	0.4746c	0.4575d	0.493c	0.5321c	0.8393a	<0.0001***	<0.0001***	<0.0001***
CA	4.31	0.2894e	2.7619c	2.9135c	4.045b	3.0992c	4.6516a	3.7101b	4.0164b	<0.0001***	<0.0001***	<0.0001***
SA	4.49	0.0836b	0.1061a	0.0438d	0.0422d	0.0896b	0.0767b	0.0709c	0.0723c	0.0001***	<0.0001***	<0.0001***
p-HCA	5.29	4.0643d	40.8317a	36.9789b	39.9402a	37.9221b	40.8281a	35.4898b	37.7969b	<0.0001***	0.0313*	<0.0001***
TFA	5.62	2.1647d	7.5001c	10.3238b	11.9867a	10.1338b	12.3666a	9.7159b	10.5248b	<0.0001***	<0.0001***	<0.0001***
SIA	5.66	0.2231f	0.9036e	2.1923b	2.0663b	1.9249c	2.9095a	3.0401a	2.0128b	<0.0001***	<0.0001***	<0.0001***
TCA	7.18	0.022 g	1.7987f	4.7117e	6.2541d	8.5254c	6.9642d	6.5519d	8.2039c	0.0002***	<0.0001***	<0.0001***
**Total phenolic acids**	−	7.7994	59.6545	59.8869	68.12	64.4285	71.1825	61.8303	66.9222	−	−	−
**Flavan-3-ols**												
CAT	3.39	ND	ND	ND	ND	ND	ND	ND	ND	−	−	−
EC	4.8	0.0018c	0e	0.005a	0e	0.0017c	0.0059a	0.0011d	0.0015c	<0.0001***	<0.0001***	<0.0001***
**Total flavan-3-ols**	−	0.0018	0	0.005	0	0.0017	0.0059	0.0011	0.0015	−	−	−

Values are expressed as µg g⁻^1^ dm. Different letters within a column indicate significant differences among samples. T, temperature; L, LED treatment; ns, not significant; *, p < 0.05; **, p < 0.01; ***, p < 0.001. Note: ND, not detected. GA, gallic acid; 4-HBA, 4-hydroxybenzoic acid; VA, vanillic acid; CA, caffeic acid; SA, syringic acid; p-HCA, p-hydroxycinnamic acid; TFA, *trans*-ferulic acid; SIA, sinapic acid; TCA, *trans*-cinnamic acid; CAT, catechin; EC, epicatechin.

**Table 4b:** (Part B). Effects of LED irradiation and ultrasound-assisted far-infrared drying on the phenolic profile of goji berries.

**Compounds**	**RT**	**Concentration (µg g⁻^1^ dm)**								**ANOVA**		
	**(min)**	**Fresh**	**Sun-dried**	**US-YL70**	**US-YL80**	**US-YL90**	**US-RL70**	**US-RL80**	**US-RL90**	**T**	**L**	**T × L**
**Flavonols**												
RUT	5.56	15.9934f	75.1124a	46.7477c	48.353c	42.4285d	36.8459e	36.2595e	32.5764e	0.0002***	<0.0001***	<0.0001***
Q3G	5.71	0.1134e	0.4417a	0.1684d	0.181d	0.204c	0.2268c	0.2068c	0.208c	<0.0001***	<0.0001***	<0.0001***
K3G	6.06	0.0288b	0.3582a	0.0217c	0.0312b	0.0231b	0.0261b	0.0404b	0.041b	<0.0001***	<0.0001***	<0.0001***
QUE	7.03	0.0194f	0.1201b	0.0673d	0.081c	0.0883c	0.0481e	0.147a	0.0696d	<0.0001***	<0.0001***	<0.0001***
KAE	7.62	0.0093c	0.0402a	0.0108c	0.0072d	0.0083d	0.0061d	0.0125c	0.0108c	<0.0001***	0.0011**	<0.0001***
ISR	7.79	0.0059d	0.0171a	0.0065d	0.0128b	0.0121b	0.006d	0.0143b	0.0106c	<0.0001***	0.3046 ns	<0.0001***
**Total flavanols**		16.1702	76.0897	47.0224	48.6662	42.7643	37.159	36.6805	32.9164	−	−	−
**Flavones**												
LUT	7.01	0.0133b	0.0164a	0.0145a	0.0052d	0.0064d	0.0086c	0.0124b	0.0126b	0.0013**	<0.0001***	<0.0001***
API	7.53	0.0025d	0.0227b	0.0044d	0.0067c	0.0036d	0.0031d	0.0623a	0.0039d	<0.0001***	<0.0001***	<0.0001***
**Total flavones**	−	0.0158	0.0391	0.0189	0.0119	0.01	0.0117	0.0747	0.0165	−	−	−
**Flavanone**												
NAR	7.53	0.111d	0.2887b	0.2536b	0.24c	0.3124a	0.2886b	0.3504a	0.3133a	−	−	−
**Total flavanone**	−	0.111	0.2887	0.2536	0.24	0.3124	0.2886	0.3504	0.3133	−	−	−
**Total flavonoids**	−	16.2988	76.4175	47.2963	48.9194	43.0895	37.4626	37.1077	33.2462	−	−	−
**Total phenolics**	−	24.0982	136.072	124.9686	121.7646	99.1714	106.1477	93.9077	92.641	−	−	−

Values are expressed as µg g⁻^1^ dm. Different letters within a column indicate significant differences among samples. T, temperature; L, LED treatment; ns, not significant; *, p < 0.05; **, p < 0.01; ***, p < 0.001. Note: RUT, rutin; Q3G, quercetin-3-O-glucoside; K3G, kaempferol-3-O-glucoside; QUE, quercetin; KAE, kaempferol; ISR, isorhamnetin; LUT, luteolin; API, apigenin; NAR, naringenin.

### 3.8 Relative PPO/POD responses and browning index

Relative PPO, relative POD, and browning index (BI) differed significantly with drying temperature, LED treatment, and their interaction (T × L) (p < 0.001; Table 3). Fresh berries were recorded as 0.00% for PPO and POD in the relative-activity dataset, whereas sun drying produced relative PPO and POD values of 142.37 and 9.98%, respectively, with BI increasing from 0.03 to 0.21. These values are reported as treatment-level relative responses and are interpreted together with BI as separate measured endpoints rather than as absolute enzyme activities. In the ultrasound-control series, relative PPO values increased from 166.57% in US-Con70 to 330.81% in US-Con80 and then decreased to 196.50% in US-Con90. Relative POD followed the same numerical pattern, increasing from 215.26% in US-Con70 to 748.92% in US-Con80 before decreasing to 303.33% in US-Con90. BI varied from 0.11 to 0.18 across these three conditions. Thus, the control series showed a clear temperature-associated change in the reported PPO/POD responses and BI, although the endpoint measurements do not establish the kinetic basis of those changes [[43], [44]]. Among the blue-LED groups, relative PPO values were 253.26, 267.35, and 279.01% at 70, 80, and 90 °C, respectively, while relative POD values were 391.78, 781.80, and 407.05%. US-BL80 therefore showed the highest relative POD response observed in the complete dataset. The corresponding BI values increased from 0.17 in US-BL70 to 0.25 in US-BL80 and 0.35 in US-BL90. Yellow-LED treatments showed relative PPO values of 314.58, 271.43, and 489.31% and relative POD values of 593.74, 717.61, and 532.09% at 70, 80, and 90 °C, respectively. Their BI values increased from 0.20 to 0.35 and 0.47 across the same temperature series. Red-LED treatments showed a different endpoint pattern: relative PPO increased from 238.48% in US-RL70 to 700.10% in US-RL80 and remained comparatively high at 562.78% in US-RL90, whereas relative POD values were lower, ranging from 91.00 to 222.50%. BI was also highest in the red-LED series, increasing from 0.23 in US-RL70 to 0.61 in US-RL80 before decreasing to 0.48 in US-RL90. These treatment-wise comparisons demonstrate that the reported PPO, POD, and BI responses did not vary in parallel across all conditions. The high BI of US-RL80 occurred concurrently with the highest relative PPO response, whereas the highest relative POD response occurred in US-BL80. This pattern supports discussion of statistical association among the measured endpoints but does not identify PPO, POD, or any non-enzymatic reaction as the sole cause of the observed browning [[45], [46], [47]]. Accordingly, the present section is restricted to treatment-dependent differences in the reported relative PPO/POD responses and BI, without assigning the observed patterns to a specific light-regulated pathway, enzyme-inactivation mechanism, or browning reaction sequence.

### 3.9 Phenolic profile

The individual phenolic profile presented in Table 4 (Parts A and B) showed substantial treatment-dependent differences in the concentrations of the quantified phenolic compounds after LED treatment and ultrasound-assisted far-infrared drying. Two-way ANOVA showed significant effects of drying temperature (T), LED treatment (L), and their interaction (T × L) for most quantified phenolics, mainly at p < 0.001, demonstrating that the endpoint phenolic profile differed across combinations of nominal LED treatment condition and drying temperature. Fresh berries contained 24.10 µg g⁻^1^ dm total quantified phenolics, whereas the sun-dried samples contained 136.07 µg g⁻^1^ dm. The higher value in the sun-dried samples was accompanied by marked concentrations of p-hydroxycinnamic acid, rutin, and *trans*-ferulic acid [17]. These values describe measured endpoint composition and are not used here as a mass-based measure of phenolic retention. In the ultrasound-control series, total quantified phenolics were 59.89, 68.12, and 64.43 µg g⁻^1^ dm in US-Con70, US-Con80, and US-Con90, respectively. Thus, the highest measured total within this control series occurred at 80 °C, while the value at 90 °C was lower. Similar changes in measured phenolic concentration with drying severity have been reported in dried fruit matrices [[18], [22]]. Ultrasound processing can affect the accessibility of matrix-associated compounds [[11], [48]]; however, because the same ultrasound step was applied to all controlled drying groups, the present between-group comparisons do not isolate an independent ultrasound contribution. The observed differences are therefore discussed as endpoint compositional responses of the complete treatment conditions rather than as evidence of a specific release, degradation, transformation, or leaching process.

Phenolic acids represented the dominant phenolic fraction. p-Hydroxycinnamic acid remained the major phenolic acid across many processed samples, and *trans*-ferulic acid, *trans*-cinnamic acid, caffeic acid, and related hydroxycinnamic derivatives also showed pronounced treatment-dependent concentration differences. For example, p-HCA was high in US-BL70 (40.83 µg g⁻^1^ dm) and US-Con80 (39.94 µg g⁻^1^ dm), while *trans*-cinnamic acid reached its highest reported concentration in US-YL80 (11.16 µg g⁻^1^ dm). Trans-ferulic acid was also prominent across several processed groups, including the blue- and yellow-LED treatments. These patterns demonstrate that individual hydroxycinnamic acids did not respond uniformly to the processing conditions. Literature on ultrasound-assisted processing provides biochemical context for changes in compound accessibility [40], but the present endpoint concentration data do not distinguish among release from the matrix, chemical conversion, degradation, or other stage-specific processes. The significant T × L responses for many compounds likewise indicate that their measured concentrations differed across treatment-temperature combinations; they are not interpreted as evidence of an isolated wavelength effect. US-YL70 showed the highest total phenolic-acid concentration among the processed groups (77.67 µg g⁻^1^ dm), whereas several red-LED groups showed lower values. Treatment-associated differences in phenolic composition following postharvest light exposure have also been reported elsewhere [49]. In the present study, however, these findings are interpreted at the compositional level and are not used as direct evidence of altered phenylpropanoid-pathway activity. US-RL90 contained 92.64 µg g⁻^1^ dm total quantified phenolics and also showed a comparatively high BI; the concurrence of these endpoints is noted without assigning the lower phenolic concentration to a defined oxidative-consumption pathway.

Flavonols formed the second major phenolic group, with rutin as the dominant compound. Rutin was highest in the sun-dried samples and remained one of the most abundant quantified flavonols across the processed treatments. Among the LED-treated groups, its concentration was higher in US-YL80 (48.35 µg g⁻^1^ dm) and US-BL90 than in US-RL90 (32.58 µg g⁻^1^ dm), showing that rutin concentration varied with treatment condition and drying temperature. Previous LC-MS studies have likewise identified rutin as a major phenolic constituent of Lycium barbarum [[1], [50]]. Minor flavonoids did not follow the same pattern. Quercetin, apigenin, naringenin, kaempferol-3-O-glucoside, quercetin-3-O-glucoside, and isorhamnetin showed compound-specific differences across the treatment combinations. For example, selected red-LED conditions showed comparatively high values for quercetin, apigenin, or naringenin even though their total quantified phenolics and rutin values were lower than those of several blue- or yellow-LED conditions. The LED main effect for isorhamnetin was not significant, whereas significant T × L responses were observed for several other flavonols, indicating that compounds within the same subclass did not respond uniformly. Taken together, the phenolic-acid and flavonoid data show that processing altered the measured phenolic profile in a compound-specific manner rather than producing a uniform change across all analytes. Differences in compound structure and processing stability can contribute to such divergent profiles [[47], [51], [52]], but the present endpoint measurements do not establish specific mechanisms such as deglycosylation, interconversion, oxidation, or conversion between bound and free phenolic forms. The phenolic results are therefore interpreted as treatment-dependent compositional differences based on the quantified endpoint concentrations.

### 3.10 Surface morphology and wax-like features

The SEM micrographs in Fig. 4a, Fig. 4b showed clear treatment-dependent differences in the endpoint external surface morphology of goji berries after processing. The fresh sample exhibited a relatively compact epidermal surface with dense granular and wax-like features, visually similar to epicuticular deposits described for fruit tissues [[53], [54]]. The sun-dried sample showed visible shrinkage and a comparatively compact surface with only limited openings [55]. Within the ultrasound-treated control series, US-Con70 displayed moderate roughness and fine fissures, whereas US-Con80 showed more pronounced folds, cavities, and open surface regions. US-Con90 appeared comparatively smoother and denser, illustrating that the endpoint surface appearance varied with the drying condition even within the common ultrasound-treated control series. These differences are consistent with the range of surface alterations reported for dried and ultrasound-treated horticultural tissues [[56], [57], [58]], but the present images are interpreted descriptively rather than as quantitative evidence of changes in cuticular permeability or internal mass transfer. The LED-treated samples also exhibited distinct endpoint morphologies. US-BL70 showed localized surface openings and irregularities, while US-BL80 and US-BL90 displayed more extensive fractured layers, enlarged openings, and fragmented regions. The US-YL70 and US-YL80 surfaces contained broad irregular and open areas, whereas US-YL90 showed a mixture of disrupted and relatively compact regions. In the red-LED series, US-RL70 and US-RL80 were characterized by comparatively smoother or locally fused-looking areas, while US-RL90 retained heterogeneous surface features with visible openings. The concurrence of these morphological differences with treatment-dependent drying and quality responses is useful for cross-comparison among the samples; however, the endpoint SEM images do not establish that the visible features caused the observed drying behaviour or biochemical changes. Likewise, terms such as internal porosity, pore connectivity, case hardening, and oxidation-driven restructuring cannot be assigned from external endpoint micrographs alone. The granular material is therefore described as wax-like on the basis of appearance only, without assigning chemical identity or barrier function. Overall, the SEM results provide a detailed comparative record of endpoint external surface morphology across the control and LED-treated samples while avoiding mechanistic interpretation that would require cross-sectional, time-resolved, or quantitative structural measurements.

**Fig. 4a:**
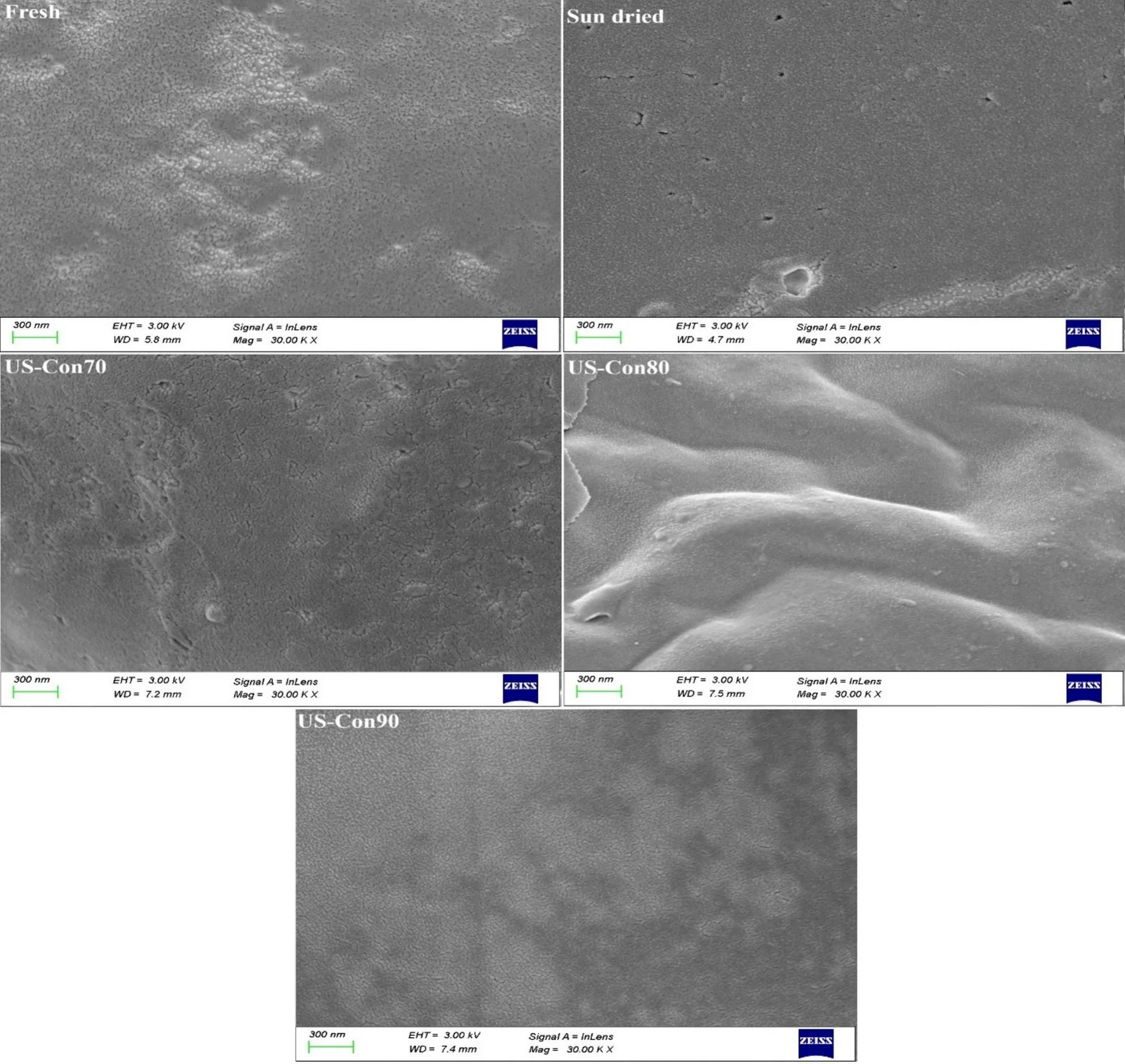
SEM micrographs of fresh, sun-dried, and ultrasound-assisted control goji berries dried at different temperatures.

**Fig. 4b:**
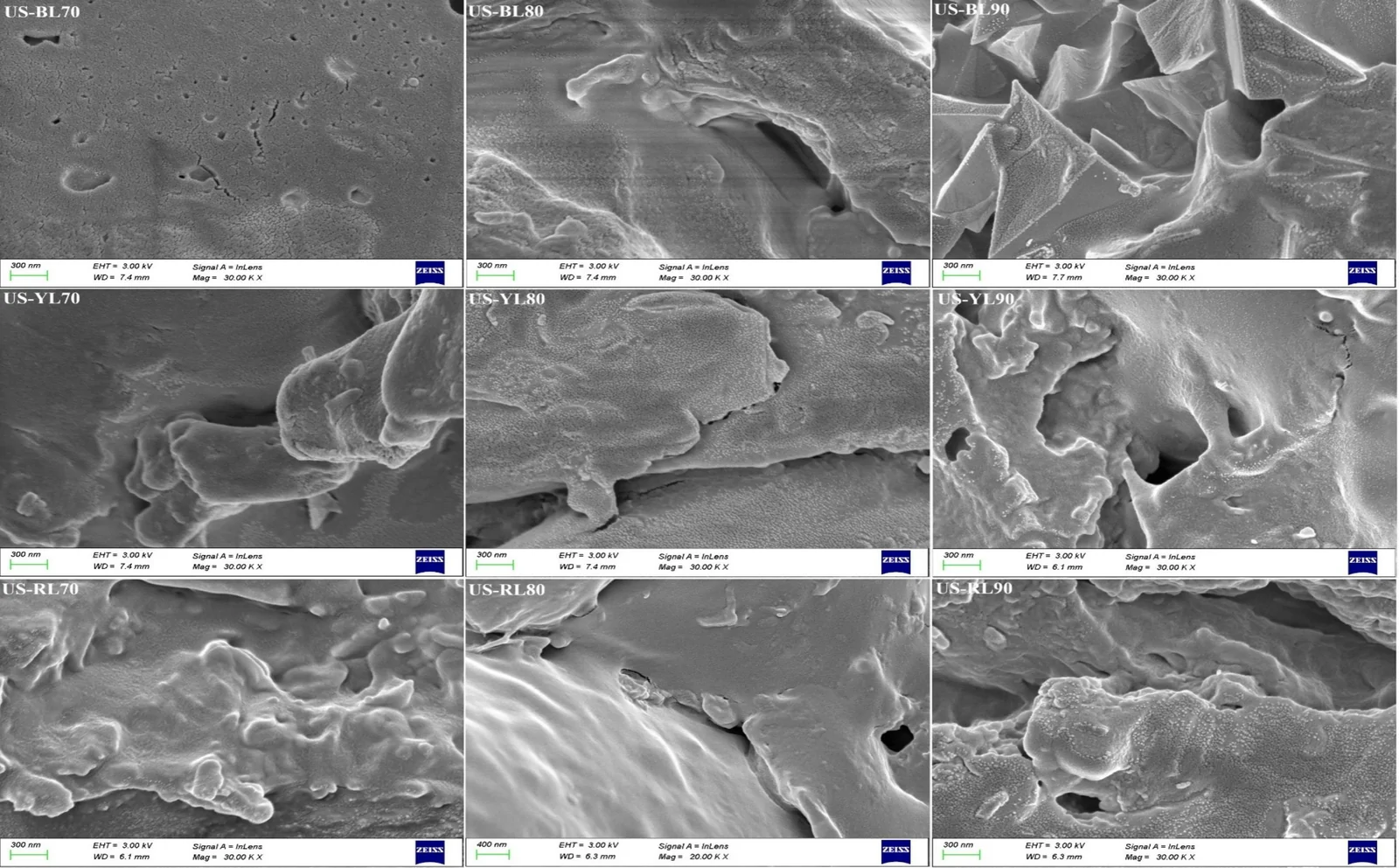
SEM micrographs of LED-treated, ultrasound-pretreated goji berries dried at different temperatures.

### 3.11 Volatile compound profile

HS-SPME/GC–MS profiling yielded 82 volatile compounds assigned across the analyzed goji berry samples, including acids, alcohols, aldehydes, ketones, esters, ethers, hydrocarbons, phenols, sulfur compounds, nitrogen/sulfur heterocycles, amides, and other minor volatiles, as listed in Supplementary Tables S4 and S5, while the Z-score heatmap in Fig. 5 summarized their treatment-specific distribution. Fresh berries were mainly characterized by alcohols, sulfur compounds, and selected esters, particularly 3-heptanol, 6-methyl-, butane, 1,1′-sulfonylbis-, and valeric/hexanoic acid esters, indicating a volatile profile containing compounds previously associated with green, fatty, fruity, and sulfurous aroma characteristics. Fruit volatiles are generally derived from fatty acid, amino acid, carotenoid, and phenylpropanoid pathways, and their relative abundance is strongly affected by postharvest processing [[59], [60], [61], [62], [63], [64]]. After sun drying, the volatile profile shifted toward greater ketone prominence, especially 3-octanone, while several alcohols and esters prominent in fresh berries were lower. Similar drying-induced shifts from fresh/fruity volatiles toward oxidized ketones and aldehydes have been reported in dried fruits and vegetables, where heat, oxygen exposure, and moisture loss accelerate volatile transformation [65]. Within the ultrasound-control treatments, US-Con80 showed higher representation of 3-heptanol, 4-methyl-, whereas US-Con90 showed marked abundance of 5-ethyl-3-methylhept-1-en-4-ol, demonstrating distinct treatment-dependent alcohol profiles [[66], [67]]. Blue-light treatments, particularly US-BL80 and US-BL90, showed higher relative abundance of 3-octanone and selected alcohols, indicating a shift toward compounds commonly associated with ketonic and green/fruity aroma characteristics. Yellow-light treatments showed the broadest treatment-associated volatile profile, especially US-YL90, where alcohols, esters, ketones, sulfur compounds, and fatty acid-derived volatiles were abundant; however, this treatment should be interpreted cautiously because US-YL90 values were estimated from TIC-derived peak areas rather than MSRep/library peak-area data. Red-light treatments were more strongly associated with ketone-rich profiles, particularly 3-octanone. In the literature, ketones and aldehydes in dried fruits are commonly associated with lipid-derived pathways, while alcohols may originate from several biochemical and processing-related routes [65]. Esters, which provide fruity and waxy notes, are sensitive to thermal processing because drying can promote ester hydrolysis, volatilization, or new ester formation depending on temperature and precursor availability [[68], [69]]. In goji berries, carotenoid degradation is particularly relevant because carotenoid-derived volatiles, including ionone-related compounds, contribute floral, fruity, and sweet aroma notes; and *trans*-β-ionone was detected in selected red-light treatments [70]. Sulfur compounds and thiazole-related volatiles were also detected in selected dried samples; related compounds have been associated in the literature with roasted, pungent, nutty, and sulfurous sensory descriptors [71]. Previous studies of dried goji berries have linked processing-related changes in esters, ionone-related compounds, and fatty-acid-derived volatiles with shifts in fruity, floral, caramel, herbaceous, fatty, mushroom-like, and cooked-fruit odor characteristics [[72], [73]]. These literature associations provide context for the compositional changes observed here, but they are not used as substitutes for treatment-specific odor-activity or sensory measurements. Because odor-activity values and sensory evaluation were not determined, the GC–MS results are interpreted as treatment-dependent volatile-profile modification rather than direct evidence of improved aroma quality.

**Fig. 5:**
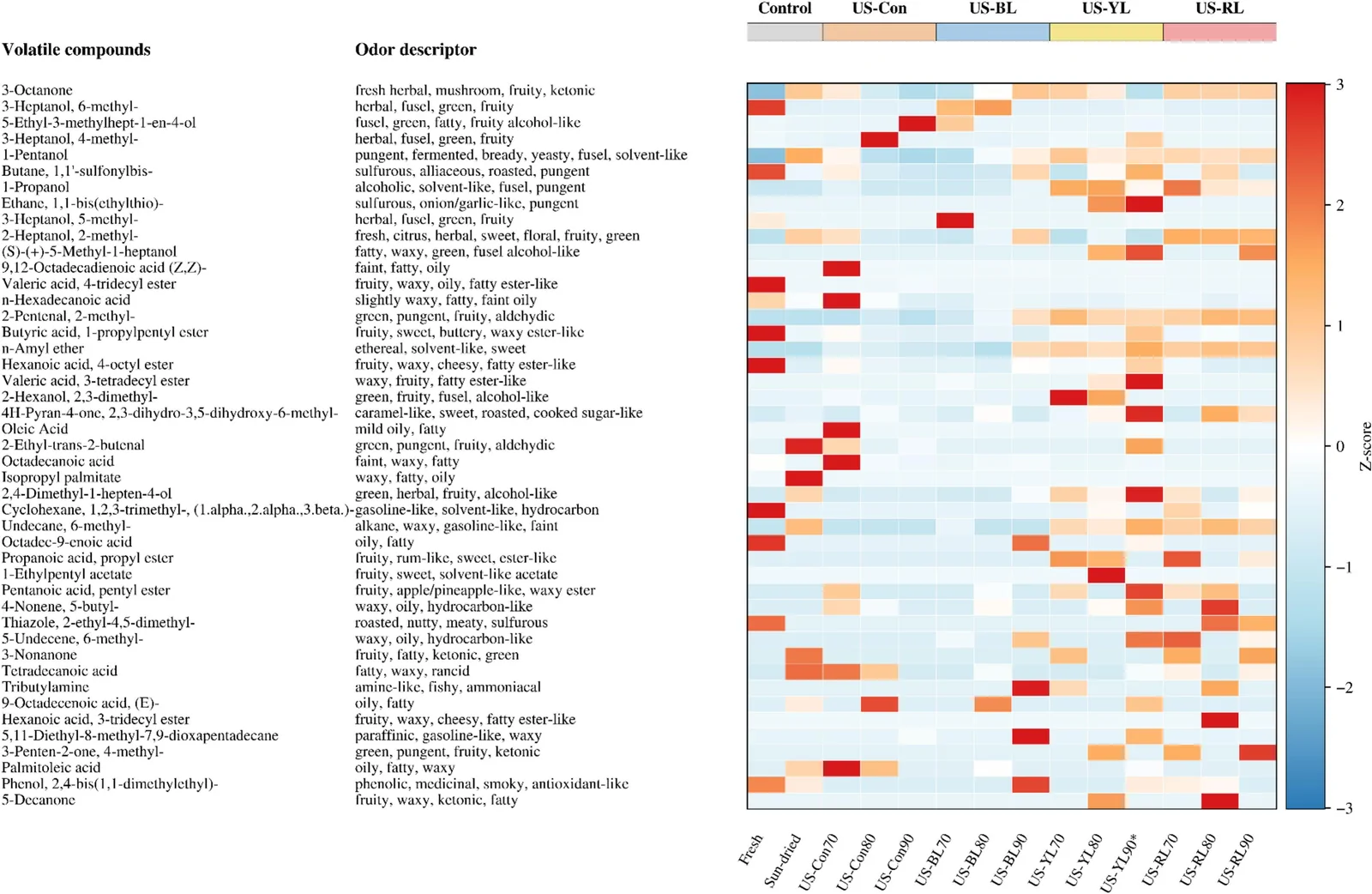
Heatmap visualization of the top volatile compounds in goji berries after LED treatment, common ultrasound pretreatment, and far-infrared drying.

### 3.12 Multivariate analysis of quality indices and phenolic compounds

The multivariate analysis presented in Fig. 6 provided an integrated statistical interpretation of the relationships among quality indices and phenolic constituents in LED-treated, ultrasound-pretreated, far-infrared-dried goji berries. PCA of the quality indices (Fig. 6a) explained 75.42% of the total variance, demonstrating that the first two components captured most of the variation among treatment conditions in DPPH, TPC, PPO, POD, and BI. Within the PCA space, PPO and BI occupied nearby positions, whereas DPPH was separated from these variables, summarizing distinct multivariate response patterns. PCA of phenolic compounds (Fig. 6b) explained 63.76% of the total variance and grouped several hydroxycinnamic acid derivatives, including p-HCA, CA, TFA, TCA, and SIA, mainly along the positive F1 region, indicating coordinated modification of phenolic acids across the evaluated treatment conditions. This pattern is consistent with the shared phenylpropanoid origin of these compounds, where structurally related phenolics often respond collectively to processing-induced stress [74]. Correlation analysis further clarified the PCA-derived relationships. In the quality-index heatmap (Fig. 6c), PPO showed a strong positive correlation with BI (r = 0.90), indicating a close statistical association between the reported PPO response and BI across the evaluated samples. Because correlation alone does not establish causality, this relationship is reported as an association only [75]. Conversely, DPPH was negatively correlated with BI (−0.32) and PPO (−0.06), indicating that higher BI and PPO values tended to occur with lower DPPH values across the evaluated samples. The weak correlation between DPPH and TPC (0.09) further shows that the two analytical endpoints did not vary in parallel across the evaluated samples. This interpretation agrees with previous findings that antioxidant activity in phenolic-rich fruits depends strongly on the molecular structure and redox behaviour of individual compounds, rather than on their total concentration [49]. In the phenolic correlation heatmap (Fig. 6d), strong positive associations among selected phenolic acids and flavonol derivatives, particularly KAE and ISR (0.85), reflected coordinated modification of structurally related compounds during drying. Similar class-dependent clustering of flavonols and hydroxycinnamic acids has been reported in berry matrices, where different phenolic groups contribute unequally to antioxidant and quality responses [76]. HCA provided an independent view of the same multivariate structure rather than repeating the PCA interpretation. In the quality-index dendrogram (Fig. 6e), PPO clustered with BI while DPPH remained relatively separated from the browning-related variables, complementing the correlation coefficients and PCA loading pattern. The phenolic dendrogram (Fig. 6f) further separated compounds into clusters broadly corresponding to phenolic acids and flavonoid derivatives, showing treatment-associated clustering within the measured phenolic dataset.

**Fig. 6:**
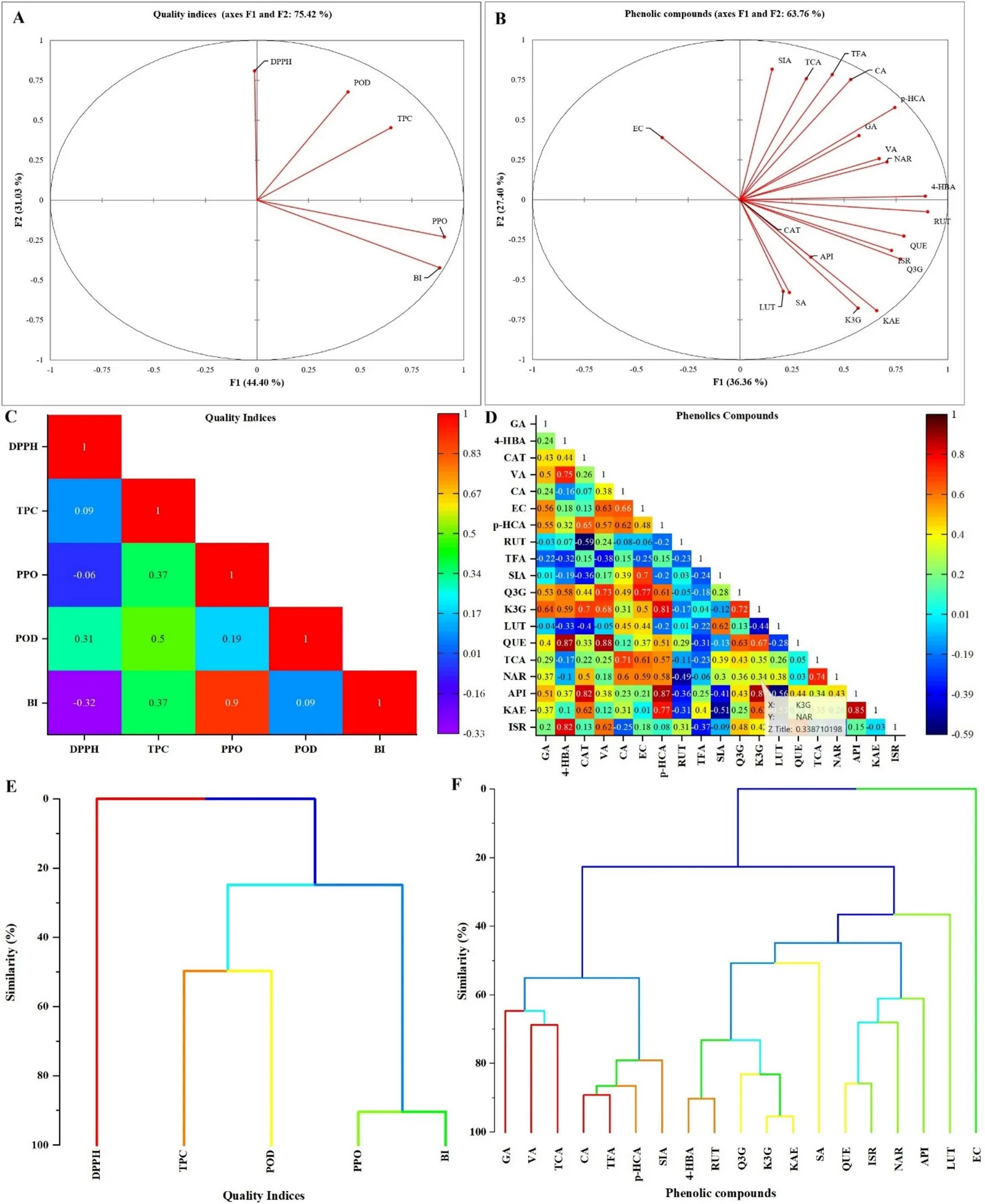
Multivariate analysis of dried goji berry quality indices and phenolic compounds: (A, B) PCA plots, (C, D) Pearson correlation heatmaps, and (E, F) HCA dendrograms, respectively.

## 4 Conclusion

This study demonstrates that LED treatment followed by a common ultrasound pretreatment produced treatment-dependent differences in far-infrared drying behaviour and the quality characteristics of goji berries. Rather than identifying a single uniformly superior treatment, the results showed treatment- and temperature-dependent differences between recorded drying behaviour and the measured quality attributes. The quality data further showed that quantified phenolic concentrations and DPPH radical-scavenging activity did not vary in parallel across all treatments; therefore, total phenolic concentration alone did not account for the observed DPPH response. Browning-related measurements were interpreted descriptively and were not used to assign a specific enzymatic or non-enzymatic mechanism. Phenolic profiling showed that US-YL70 had the highest summed concentration of the quantified phenolic compounds among the LED-treated, ultrasound-pretreated experimental groups, with treatment-specific differences also observed for individual phenolic acids and flavonoids. Volatile profiling indicated treatment-associated differences in the reported endpoint dataset; these findings were interpreted compositionally rather than as direct evidence of improved aroma quality. SEM observations showed treatment-dependent differences in endpoint external surface morphology, including openings, fissures, compact regions, and wax-like surface features, without being used to infer internal porosity, case hardening, or a specific moisture-transport mechanism. Overall, the observed differences are interpreted among the nominal LED treatment conditions within a common ultrasound-treated background and do not establish an isolated wavelength response or an LED × ultrasound interaction. Accordingly, the relative performance of each treatment depends on the specific measured attribute and drying temperature considered. The calculated dryer-stage energy estimates in this study are restricted to the far-infrared heating-and-blower boundary and do not represent the total energy demand of the complete LED-ultrasound-drying sequence. The present findings establish laboratory-scale processing responses and do not by themselves establish industrial economic competitiveness or environmental superiority.

## Funding

This work was also supported by the Deanship of Scientific Research, Vice Presidency for 10.13039/100023807Graduate Studies and Scientific Research, 10.13039/501100020912King Faisal University, Saudi Arabia [GRANT: KFU263353].

## CRediT authorship contribution statement

**Muhammad Tayyab Rashid:** Writing – original draft, Software, Methodology, Investigation, Formal analysis, Data curation, Conceptualization. **Nazish Muzaffar:** Writing – review & editing, Data curation. **Longquan Fan:** Writing – review & editing, Supervision, Resources, Methodology, Investigation, Formal analysis, Data curation, Conceptualization. **Mushtaque Ahmed Jatoi:** Writing – review & editing, Methodology. **Abderrahmane Aït-Kaddour:** Writing – review & editing, Funding acquisition. **Jasur Safarov:** . **Marwa Ezz El-Din Ibrahim:** Writing – review & editing, Methodology. **Rana Muhammad Aadil:** Writing – review & editing, Methodology.

## Declaration of competing interest

The authors declare that they have no known competing financial interests or personal relationships that could have appeared to influence the work reported in this paper.

## Appendix A Supplementary data

Supplementary Data 1 Drying-model, literature-comparison, and volatile-profile data for treated goji berries.
