# Supplementary material for: Ultrasound- and LED-mediated modulation of drying behaviour, surface morphology, phenolic composition, and volatile profiles in goji berries
Source: Ultrason Sonochem. 2026 Oct 13;134:108115. doi: 10.1016/j.ultsonch.2026.108115 (PMC13696815; doi:10.1016/j.ultsonch.2026.108115)
Supplement: Supplementary file 1 [file mmc1.docx]

**Supplementary Table S1. Focused comparison of closely related goji drying studies and the scientific positioning of the current study.**

.

| **Study focus** | **Processing design** | **Drying system** | **Main analytical coverage** | **Relationship to the present study** | **Reference** |
| --- | --- | --- | --- | --- | --- |
| Dewaxing + hybrid drying | Sodium-carbonate dewaxing combined with hybrid drying. | FD-DIC and hot-air drying/DIC, with individual-method comparisons. | Microstructure, physicochemical quality, polysaccharides, carotenoids, ABTS activity. | Shows that pretreatment and hybrid drying can alter goji structure and composition. It did not examine LED exposure, a common ultrasound step, or FIR drying, so it provides supporting context rather than a direct precedent. | [1] |
| Ultrasound-assisted FIR drying | Ultrasound conditions were varied before/during FIR drying of wolfberry. | Ultrasound-assisted FIR drying; frequency, power, irradiation height, and temperature were evaluated. | Drying characteristics, selected quality indices, antioxidant-related measures, microstructure, thin-layer models. | Closest process precedent; confirms that ultrasound-assisted FIR drying of wolfberry is not novel itself. The present study instead evaluates nominal LED conditions within a common ultrasound/FIR sequence and adds broader phenolic and volatile profiling. | [2] |
| Ultrasound-associated drying routes | Conventional and ultrasound-associated drying methods were compared. | Hot-air, vacuum, ultrasound-assisted vacuum, freeze-drying, and ultrasound-pretreated freeze-drying. | Drying kinetics, TPC, DPPH/CUPRAC, individual phenolics, carotenoids, colour. | Demonstrates that ultrasound-associated drying can modify dehydration and quality endpoints in goji berry. It did not include LED treatment or the specific LED → common ultrasound → FIR sequence used here. | [3] |
| Ripening stage during drying | Goji berries at different ripening stages were dried and compared. | Pulsed-vacuum drying. | Ultrastructure, drying behaviour, moisture status, physicochemical quality, browning. | Shows that initial fruit state can influence later drying and browning responses. The factor was ripening stage, not LED treatment, and no common ultrasound/FIR sequence was used. | [4] |
| Current study | Nominal blue-, yellow-, red-LED and non-irradiated groups, all followed by the same ultrasound pretreatment. | FIR drying at 70, 80, and 90 °C chamber setpoints. | Drying behaviour, activation descriptors, DPPH, TPC, relative PPO/POD, browning, phenolics, SEM, HS-SPME/GC-MS volatiles. | Integrates nominal LED conditions with a common ultrasound/FIR sequence across three drying setpoints and multiple endpoint analyses. It does not claim an isolated wavelength effect, an independent ultrasound effect, or an LED × ultrasound interaction. | Current study |

**Supplementary Table S2**. Drying models used to study drying kinetics of ultrasound and LED-treated goji berries

| **S. No** | **Model name** | **Model** | **References** |
| --- | --- | --- | --- |
| 1 | Newton | $Mr=exp(-kt)$ | [5] |
| 2 | Page | $Mr=exp(-kt^{n}$) | [6] |
| 3 | Handerson and Pabis model | $Mr=aexp(-kt^{n}$) | [7] |
| 4 | Modified Henderson and Pabis | $Mr=aexp\left( -kt \right)+bexp\left( -gt \right)+cexp(-ht)$ | [8] |
| 5 | Logarithmic model | $Mr=aexp\left( -kt \right)+c$ | [9] |
| 6 | Midilli model | $Mr=aexp\left( -kt \right)+bt$ | [10] |
| 7 | Two-term model | $Mr=aexp\left( {-k}_{1}t \right)+bexp\left( {-k}_{2}t \right)$ | [11] |
| 8 | Hii model | $Mr=aexp\left( {-k}_{1}t^{n} \right)+bexp\left( {-k}_{2}t^{n} \right)$ | [12] |
| 9 | Verma model | $Mr=aexp\left( -kt \right)+\left( 1-a \right)exp(-gt)$ | [13] |
| 10 | Modified Midilli model | $Mr=aexp\left( -kt \right)+b$ | [14] |
| 11 | Aghbashlo model | $Mr=exp\left( k_{1}t/1+k_{2}t \right)$ | [15] |
| 12 | Wang and Singh | $Mr=1+at+bt^{2}$ | [16] |
| 13 | Silva model | $Mr=exp(-at-b\sqrt{t})$ | [17] |

**Supplementary Table S3.** Averages of selected models fitted to the ultrasound and LED-treated treated goji berries.

| **Sample Codes** | **Coefficients** | **R^2^** | **RSS** | **χ^2^** | **RMSE** |
| --- | --- | --- | --- | --- | --- |
|  | **1, Modified Henderson and Pabis model** |  |  |  |  |
| US-Con1  US-Con2  US-Con3  US-BlueL1  US-BlueL2  US-BlueL3  US-YellowL4  US-YellowL5  US-YellowL6  US-RedL7  US-RedL8  US-RedL9 | a. 0.796, k. 0.005, b. 0.163, g. 0.005, c. 0.163, h, 0.005  a. 0.532, k. 0.008, b. 0.689, g. 0.469, c. 0.138, h, 0.005  a. 0.710, k. 0.005, b. 0.199, g. 0.005, c. 0.199, h, 0.005  a. 2.826, k. 0.003, b. 0.891, g. 0.002, c. 0.089, h, 0.002  a. 2.785, k. 0.004, b. 0.869, g. 0.003, c. 0.869, h, 0.003  a. 0.897, k. 0.012, b. 0.075, g. 0.012, c. 0.075, h, 0.012  a. 2.640, k. 0.004, b. 0.797, g. 0.003, c. 0.797, h, 0.003  a. 2.926, k. 0.004, b. 0.927, g. 0.003, c. 0.927, h, 0.003  a. 0.959, k. 0.012, b. 0.055, g. 0.012, c. 0.055, h, 0.012  a. 2.619, k. 0.004, b. 0.777, g. 0.003, c. 0.777, h, 0.003  a. 2.631, k. 0.005, b. 0.780, g. 0.003, c. 0.780, h, 0.003  a. 1.887, k. 0.005, b. 0.719, g. 0.005, c. 1.548, h, 0.004 | 0.987  0.960  0.960  0.993  0.991  0.982  0.988  0.986  0.981  0.986  0.985  0.987 | 0.04  0.10  0.01  0.01  0.02  0.00  0.03  0.03  0.00  0.03  0.03  0.01 | 0.0035  0.0110  0.0151  0.0013  0.0022  0.0022  0.0028  0.0029  0.0070  0.0026  0.0026  0.0040 | 0.0369  0.0658  0.0674  0.0272  0.0314  0.0420  0.0359  0.0408  0.0448  0.0404  0.0409  0.0387 |
|  | **2, Two-term model** |  |  |  |  |
| US-Con1  US-Con2  US-Con3  US-BlueL1  US-BlueL2  US-BlueL3  US-YellowL4  US-YellowL5  US-YellowL6  US-RedL7  US-RedL8  US-RedL9 | a. 15.511, k1. 0.001, b. 15.455, k2. 0.001  a. 0.919, k1. 0.005, b. 0.202, k2. 0.005  a. 0.811, k1. 0.005, b. 0.297, k2. 0.005  a. 24.326, k1. 0.003, b. 23.281, k2. 0.003  a. 27.638, k1. 0.004, b. 26.591, k2. 0.004  a. 58.492, k1. 0.020, b. 57.496, k2. 0.020  a. 27.616, k1. 0.003, b. 26.570, k2. 0.003  a. 33.432, k1. 0.004, b. 32.361, k2. 0.004  a. 75.705, k1. 0.021, b. 74.711, k2. 0.022  a. 34.723, k1. 0.004, b. 33.658, k2. 0.004  a. 33.464, k1. 0.004, b. 32.393, k2. 0.004  a. 36.615, k1. 0.005, b. 35.558, k2. 0.005 | 0.992  0.960  0.960  0.993  0.992  0.991  0.991  0.986  0.998  0.986  0.985  0.988 | 0.03  0.10  0.09  0.01  0.02  0.01  0.03  0.03  0.09  0.03  0.03  0.02 | 0.0024  0.0110  0.0151  0.0013  0.0022  0.0011  0.0027  0.0029  0.0006  0.0025  0.0025  0.0040 | 0.0313  0.0658  0.0674  0.0272  0.0314  0.0299  0.0354  0.0408  0.0135  0.0395  0.0399  0.0387 |
|  | **3, Modified Midilli** |  |  |  |  |
| US-Con1  US-Con2  US-Con3  US-BlueL1  US-BlueL2  US-BlueL3  US-YellowL4  US-YellowL5  US-YellowL6  US-RedL7  US-RedL8  US-RedL9 | a. 0.756, k. 0.034, n. 0.546, b. 0.645,  a. 0.453, k. 0.065, n. 0.654, b. 0.751,  a. 0.234, k. 0.023, n. 0.756, b. 0.346,  a. 0.476, k. 0.087, n. 0.234, b. 0.567,  a. 0.632, k. 0.034, n. 0.787, b. 0.534,  a. 0.587, k. 0.098, n. 0.235, b. 0.867,  a. 0.823, k. 0.034, n. 0.654, b. 0.756,  a. 0.298, k. 0.087, n. 0.676, b. 0.657,  a. 0.356, k. 0.045, n. 0.867, b. 0.723,  a. 0.199, k. 0.076, n. 0.456, b. 0.756,  a. 0.734, k. 0.065, n. 0.365, b. 0.213,  a. 0.843, k. 0.032, n. 0.734, b. 0.645, | 0.87  0.86  0.84  0.85  0.88  0.82  0.83  0.87  0.88  0.85  0.88  0.86 | 7.10  5.31  4.53  3.96  3.20  2.20  3.71  3.29  2.31  3.32  3.11  2.88 | 0.6450  0.5899  0.7543  0.3601  0.4575  0.1998  0.4123  0.3653  0.5772  0.3014  0.3105  0.4794 | 0.5126  0.4804  0.4757  0.4450  0.4473  0.3962  0.4307  0.4533  0.4061  0.4292  0.4405  0.4240 |
|  | **4, Verma Model** |  |  |  |  |
| US-Con1  US-Con2  US-Con3  US-BlueL1  US-BlueL2  US-BlueL3  US-YellowL4  US-YellowL5  US-YellowL6  US-RedL7  US-RedL8  US-RedL9 | a. 14.814, k. 0.001, g. 0.001  a. 17.560, k. 0.001, g. 0.001  a. 15.881, k. 0.002, g. 0.002  a. 17.831, k. 0.002, g. 0.003  a. 19.370, k. 0.003, g. 0.003  a. 22.173, k. 0.006, g. 0.007  a. 20.668, k. 0.003, g. 0.003  a. 22.454, k. 0.003, g. 0.003  a. 30.252, k. 0.006, g. 0.006  a. 23.057, k. 0.003, g. 0.003  a. 23.957, k. 0.004, g. 0.004  a. 25.328, k. 0.004, g. 0.004 | 0.998  0.987  0.989  0.991  0.989  0.989  0.987  0.981  0.989  0.982  0.980  0.984 | 0.03  0.03  0.02  0.02  0.02  0.02  0.03  0.04  0.01  0.04  0.03  0.03 | 0.0029  0.0037  0.0039  0.0017  0.0025  0.0014  0.0031  0.0041  0.0037  0.0032  0.0033  0.0044 | 0.0349  0.0380  0.0345  0.0306  0.0331  0.0334  0.0375  0.0485  0.0326  0.0442  0.0457  0.0406 |
|  | **5, Henderson and Pabis model** |  |  |  |  |
| US-Con1  US-Con2  US-Con3  US-BlueL1  US-BlueL2  US-BlueL3  US-YellowL4  US-YellowL5  US-YellowL6  US-RedL7  US-RedL8  US-RedL9 | a. 0.451, k. 0.017, n. 0.543,  a. 0.234, k. 0.053, n. 0.676  a. 0.275, k. 0.087, n. 0.456  a. 0.867, k. 0.021, n. 0.213  a. 0.234, k. 0.045, n. 0.756  a. 0.867, k. 0.065, n. 0.587  a. 0.217, k. 0.076, n. 0.756  a. 0.867, k. 0.078, n. 0.545  a. 0.678, k. 0.023, n. 0.578  a. 0.645, k. 0.014, n. 0.127  a. 0.124, k. 0.018, n. 0.756  a. 0.756, k. 0.076, n. 0.678 | 0.782  0.771  0.750  0.767  0.796  0.724  0.742  0.783  0.790  0.764  0.791  0.772 | 7.10  5.31  4.53  3.96  3.20  2.20  3.71  3.29  2.31  3.32  3.11  2.88 | 0.6450  0.5899  0.7543  0.3601  0.4575  0.1998  0.4123  0.3653  0.5772  0.3014  0.3105  0.4794 | 0.5126  0.4804  0.4757  0.4450  0.4473  0.3962  0.4307  0.4533  0.4061  0.4292  0.4405  0.4240 |
|  | **8, Logarithmic model** |  |  |  |  |
| US-Con1  US-Con2  US-Con3  US-BlueL1  US-BlueL2  US-BlueL3  US-YellowL4  US-YellowL5  US-YellowL6  US-RedL7  US-RedL8  US-RedL9 | a. 1.577, k. 0.002, n. 5.22  a. 1.334, k. 0.003, n. 0.274  a. 1.329, k. 0.003, n. 0.281  a. 1.195, k. 0.004, n. 0.147  a. 1.191, k. 0.006, n. 0.141  a. 1.080, k. 0.010, n. 0.050  a. 1.166, k. 0.005, n. 1.116  a. 1.216, k. 0.006, n. 0.143  a. 1.111, k. 0.010, n. 0.059  a. 1.174, k. 0.006, n. 0.106  a. 1.184, k. 0.007, n. 0.111  a. 1.147, k. 0.007, n. 0.086 | 0.991  0.989  0.990  0.991  0.990  0.988  0.987  0.983  0.988  0.983  0.982  0.985 | 0.03  0.03  0.02  0.02  0.02  0.02  0.03  0.03  0.02  0.03  0.03  0.02 | 0.0024  0.0031  0.0038  0.0017  0.0025  0.0015  0.0031  0.0032  0.0047  0.0030  0.0031  0.0041 | 0.0315  0.0350  0.0339  0.0308  0.0333  0.0348  0.0377  0.0430  0.0369  0.0429  0.0442  0.0394 |
|  | **6, Aghbashlo Model** |  |  |  |  |
| US-Con1  US-Con2  US-Con3  US-BlueL1  US-BlueL2  US-BlueL3  US-YellowL4  US-YellowL5  US-YellowL6  US-RedL7  US-RedL8  US-RedL9 | k1. 1.765, k2. 1.768  k1. 2.260 k2. 2.256  k1. 2.532 k2. 2.527  k1. 2.814 k2. 2.808  k1. 5.476 k2. 5.469  k1. 8.889 k2. 8.901  k1. 4.077 k2. 4.083  k1. 3.880 k2. 3.887  k1. 9.469 k2. 9.480  k1. 6.303 k2. 6.310  k1. 7.562 k2. 7.570  k1. 7.769 k2. 7.778 | 0.932  0.945  0.948  0.966  0.965  0.980  0.965  0.953  0.976  0.959  0.957  0.966 | 0.21  0.14  0.12  0.07  0.06  0.03  0.08  0.09  0.03  0.08  0.08  0.06 | 0.0187  0.0157  0.0199  0.0065  0.0086  0.0247  0.0084  0.0100  0.0087  0.0075  0.0080  0.0094 | 0.0874  0.0785  0.0772  0.0600  0.0616  0.0702  0.0616  0.0750  0.0498  0.0677  0.0708  0.0595 |
|  | **7, Wang and Singh** |  |  |  |  |
| US-Con1  US-Con2  US-Con3  US-BlueL1  US-BlueL2  US-BlueL3  US-YellowL4  US-YellowL5  US-YellowL6  US-RedL7  US-RedL8  US-RedL9 | a. 0.002, b. 9.377 ×10^-07^  a. 0.003, b. 2.116 ×10^-06^  a. 0.003, b. 2.918 ×10^-06^  a. 0.004, b. 3.945 ×10^-06^  a. 0.005, b. 6.582 ×10^-06^  a. 0.007, b. 1.241 ×10^-05^  a. 0.004, b. 4.476 ×10^-06^  a. 0.005, b. 6.730 ×10^-06^  a. 0.007, b. 1.229 ×10^-05^  a. 0.005, b. 6.071 ×10^-06^  a. 0.006, b. 7.470 ×10^-06^  a. 0.006, b. 8.143 ×10^-06^ | 0.991  0.992  0.995  0.998  0.998  0.978  0.995  0.993  0.988  0.994  0.992  0.993 | 0.03  0.02  0.01  0.00  0.00  0.03  0.01  0.01  0.02  0.01  0.01  0.01 | 0.0025  0.0022  0.0016  0.0030  0.0006  0.0025  0.0011  0.0015  0.0047  0.0010  0.0014  0.0018 | 0.0315  0.0294  0.0254  0.0146  0.0163  0.0450  0.0232  0.0296  0.0368  0.0250  0.0301  0.0266 |
|  | **8, Silva Model** |  |  |  |  |
| US-Con1  US-Con2  US-Con3  US-BlueL1  US-BlueL2  US-BlueL3  US-YellowL4  US-YellowL5  US-YellowL6  US-RedL7  US-RedL8  US-RedL9 | a. 0.005, b. 0.037  a. 0.007, b. 0.041  a. 0.008, b. 0.042  a. 0.008, b. 0.039  a. 0.011, b. 0.049  a. 0.016, b. 0.045  a. 0.009, b. 0.040  a. 0.012, b. 0.062  a. 0.017, b.0.065  a. 0.012, b. 0.055  a. 0.013, b. 0.063  a. 0.013, b. 0.057 | 0.976  0.981  0.979  0.988  0.989  0.990  0.985  0.988  0.996  0.987  0.990  0.991 | 0.07  0.05  0.05  0.02  0.02  0.02  0.03  0.02  0.01  0.03  0.02  0.02 | 0.0064  0.0053  0.0077  0.0022  0.0027  0.0014  0.0036  0.0026  0.0017  0.0022  0.0017  0.0027 | 0.0514  0.0457  0.0480  0.0351  0.0344  0.0339  0.0405  0.0384  0.0221  0.0374  0.0332  0.0320 |
|  | **9, Page model** |  |  |  |  |
| US-Con1  US-Con2  US-Con3  US-BlueL1  US-BlueL2  US-BlueL3  US-YellowL4  US-YellowL5  US-YellowL6  US-RedL7  US-RedL8  US-RedL9 | a.1.577, k. 0.002, c. 0.522  a.1.334, k. 0.003, c. 0.274  a.1.329, k. 0.003, c. 0.281  a.1.195, k. 0.004, c. 0.147  a.1.191, k. 0.006, c. 0.141  a.1.080, k. 0.010, c. 0.050  a.1.166, k. 0.005, c. 0.116  a.1.216, k. 0.006, c. 0.143  a.1.111 k. 0.010, c. 0.059  a.1.174, k. 0.006, c. 0.106  a.1.184 k. 0.007, c. 0.111  a.1.147, k. 0.007, c. 0.086 | 0.991  0.989  0.990  0.991  0.990  0.988  0.987  0.983  0.988  0.983  0.982  0.985 | 0.01  0.01  0.01  0.01  0.01  0.01  0.01  0.00  0.00  0.01  0.00  0.00 | 0.0010  0.0011  0.0024  0.0006  0.0008  0.0011  0.0015  0.0005  0.0003  0.0005  0.0003  0.0007 | 0.0210  0.0212  0.0268  0.0184  0.0191  0.0298  0.0261  0.0173  0.0093  0.0178  0.0144  0.0171 |
|  | **10, Newton** |  |  |  |  |
| US-Con1  US-Con2  US-Con3  US-BlueL1  US-BlueL2  US-BlueL3  US-YellowL4  US-YellowL5  US-YellowL6  US-RedL7  US-RedL8  US-RedL9 | k.0.003  k.0.004  k.0.005  k.0.006  k.0.007  k.0.011  k.0.006  k.0.007  k.0.011  k.0.007  k.0.008  k.0.008 | 0.932  0.945  0.948  0.966  0.965  0.980  0.965  0.953  0.976  0.959  0.957  0.966 | 0.21  0.14  0.12  0.07  0.06  0.03  0.08  0.09  0.03  0.08  0.08  0.06 | 0.0187  0.0157  0.0199  0.0065  0.0086  0.0024  0.0084  0.0100  0.0087  0.0075  0.0080  0.0094 | 0.0874  0.0785  0.0772  0.0600  0.0616  0.0440  0.0616  0.0750  0.0498  0.0677  0.0708  0.0595 |

**Supplementary Table S4. Effect of LED pretreatment and ultrasound-assisted convective drying on ng/g concentration of volatile compounds of goji berries.**

| **No.** | **Compounds** | **RT (min)** | **Fresh** | **Sun-dried** | **US-Con70** | **US-Con80** | **US-Con90** | **US-BL70** | **US-BL80** | **US-BL90** | **US-YL70** | **US-YL80** | **US-YL90** | **US-RL70** | **US-RL80** | **US-RL90** |
| --- | --- | --- | --- | --- | --- | --- | --- | --- | --- | --- | --- | --- | --- | --- | --- | --- |
| **A** | **Acids** |  |  |  |  |  |  |  |  |  |  |  |  |  |  |  |
| 1 | 6-Octadecenoic acid, (Z)- | 48.711 | - | - | - | - | 0.005 | 0.027 | - | - | - | - | 0.171 | 0.000 | - | - |
| 2 | 9,12-Octadecadienoic acid (Z,Z)- | 48.652 | - | - | 0.145 | 0.022 | 0.004 | 0.021 | 0.043 | 0.008 | - | - | 0.171 | - | - | - |
| 3 | 9-Hexadecenoic acid | 45.035 | - | - | - | - | - | - | - | - | - | - | - | - | - | 0.002 |
| 4 | 9-Octadecenoic acid, (E)- | 48.701 | - | 0.002 | - | 0.037 | - | - | 0.073 | - | - | - | 0.171 | - | - | - |
| 5 | Docosanoic acid | 57.216 | 0.006 | - | - | - | - | - | - | - | - | - | - | - | - | - |
| 6 | Octadec-9-enoic acid | 48.750 | 0.030 | - | - | - | - | - | - | 0.019 | - | - | 0.171 | - | - | - |
| 7 | Octadecanoic acid | 49.163 | 0.011 | - | 0.036 | 0.015 | 0.001 | - | 0.035 | 0.005 | - | - | 0.085 | - | - | - |
| 8 | Oleic Acid | 48.896 | - | - | 0.051 | - | - | - | - | - | - | - | 0.171 | - | - | - |
| 9 | Palmitoleic acid | 45.021 | - | 0.003 | 0.003 | 0.020 | - | - | 0.015 | - | - | - | 0.046 | - | - | - |
| 10 | Pentadecanoic acid | 43.443 | - | 0.002 | - | 0.009 | - | - | 0.009 | - | - | - | 0.043 | - | - | - |
| 11 | Tetradecanoic acid | 41.364 | - | 0.006 | 0.003 | 0.019 | - | - | 0.016 | - | - | - | 0.057 | 0.000 | - | 0.002 |
| 12 | n-Decanoic acid | 31.218 | - | 0.002 | - | - | - | - | - | - | - | - | - | - | - | - |
| 13 | n-Hexadecanoic acid | 45.444 | 0.072 | 0.017 | 0.056 | 0.088 | 0.005 | 0.020 | 0.133 | 0.011 | 0.013 | 0.001 | 0.467 | 0.001 | 0.007 | 0.008 |
| **B** | **Alcohols** |  |  |  |  |  |  |  |  |  |  |  |  |  |  |  |
| 14 | (S)-(+)-5-Methyl-1-heptanol | 18.413 | - | - | - | - | - | - | - | - | - | 0.010 | 8.456 | - | - | 0.125 |
| 15 | 1-Pentanol | 3.593 | - | 0.388 | 0.097 | 0.405 | 0.161 | 1.346 | 2.402 | 0.293 | 0.733 | 0.020 | 12.314 | 0.029 | 0.335 | 0.245 |
| 16 | 1-Propanol | 1.275 | - | - | 0.014 | 0.029 | 0.021 | 0.095 | 0.235 | 0.055 | 0.439 | 0.017 | 3.727 | 0.024 | 0.133 | 0.081 |
| 17 | 2,4-Dimethyl-1-hepten-4-ol | 17.026 | - | 0.009 | - | 0.004 | 0.003 | - | - | 0.004 | 0.020 | 0.000 | 0.914 | 0.001 | - | 0.005 |
| 18 | 2-Heptanol, 2-methyl- | 10.843 | - | 0.065 | 0.022 | 0.074 | 0.035 | 0.126 | 0.297 | 0.075 | - | 0.002 | 0.131 | 0.008 | 0.094 | 0.063 |
| 19 | 2-Hexanol, 2,3-dimethyl- | 10.828 | - | - | 0.003 | - | - | 0.029 | - | - | 0.232 | 0.005 | 0.064 | - | - | - |
| 20 | 2-Pentanol, 3-ethyl-2-methyl- | 13.068 | - | - | - | - | - | - | 0.053 | - | - | - | - | - | - | - |
| 21 | 3-Heptanol, 4-methyl- | 3.588 | 0.116 | 0.035 | - | 9.999 | 0.012 | 0.119 | - | 0.034 | 0.182 | 0.004 | 30.108 | 0.005 | 0.056 | 0.032 |
| 22 | 3-Heptanol, 5-methyl- | 3.272 | 0.112 | - | - | - | - | 7.703 | - | - | - | - | 0.574 | - | - | - |
| 23 | 3-Heptanol, 6-methyl- | 4.255 | 2.757 | - | - | - | - | 18.542 | 15.637 | - | - | - | 4.147 | - | - | - |
| 24 | 3-Nonanol | 20.701 | - | - | - | - | - | 0.009 | - | - | - | - | - | - | - | - |
| 25 | 4,5-Dimethyl-3-heptanol | 25.799 | 0.016 | - | - | - | - | - | - | - | - | - | - | - | - | - |
| 26 | 5-Ethyl-3-methylhept-1-en-4-ol | 3.496 | 0.086 | - | - | - | 9.138 | 16.451 | - | - | - | - | 1.681 | - | - | - |
| **C** | **Aldehydes** |  |  |  |  |  |  |  |  |  |  |  |  |  |  |  |
| 27 | 2-Ethyl-trans-2-butenal | 5.804 | - | 0.054 | 0.008 | - | 0.016 | - | - | - | - | - | 1.437 | - | - | - |
| 28 | 2-Pentenal, 2-methyl- | 5.711 | - | - | - | 0.031 | - | 0.105 | 0.181 | 0.036 | 0.092 | 0.003 | 1.437 | 0.003 | 0.050 | 0.033 |
| 29 | 4,9,13,17-Tetramethyl-4,8,12,16-octadecatetraenal | 43.020 | - | 0.002 | - | - | - | - | - | - | - | - | - | - | - | - |
| **D** | **Ketones** |  |  |  |  |  |  |  |  |  |  |  |  |  |  |  |
| 30 | 2(4H)-Benzofuranone, 5,6,7,7a-tetrahydro-4,4,7a-trimethyl- | 35.907 | - | 0.002 | - | - | - | - | - | - | - | - | - | - | - | 0.001 |
| 31 | 2-Cyclopenten-1-one, 2,3,4,5-tetramethyl- | 21.169 | - | 0.001 | - | - | - | - | - | - | - | - | - | - | 0.002 | - |
| 32 | 3-Nonanone | 19.431 | - | 0.008 | - | - | - | - | - | - | 0.011 | - | - | 0.001 | - | 0.005 |
| 33 | 3-Octanone | 14.134 | - | 2.347 | 0.763 | 4.523 | 1.464 | 10.776 | 18.853 | 2.793 | 4.917 | 0.148 | 24.187 | 0.215 | 2.591 | 1.811 |
| 34 | 3-Penten-2-one, 4-methyl- | 6.227 | - | - | - | - | - | - | - | - | - | 0.000 | - | 0.000 | - | 0.006 |
| 35 | 4-Nonene, 5-butyl- | 26.709 | - | - | 0.002 | 0.009 | - | - | 0.034 | - | - | 0.000 | 0.402 | - | 0.015 | - |
| 36 | 4H-Pyran-4-one, 2,3-dihydro-3,5-dihydroxy-6-methyl- | 22.001 | - | 0.004 | - | 0.040 | 0.013 | 0.021 | 0.136 | - | 0.013 | 0.001 | 2.197 | - | 0.037 | 0.015 |
| 37 | 5-Decanone | 23.628 | - | - | - | - | - | - | - | - | - | 0.000 | - | - | 0.011 | - |
| 38 | Ar-tumerone | 39.300 | - | - | - | - | - | 0.038 | - | - | - | - | - | - | - | - |
| 39 | Curlone | 40.122 | - | - | - | - | - | 0.018 | - | - | - | - | - | - | - | - |
| 40 | Tumerone | 39.382 | - | - | - | - | - | 0.019 | - | - | - | - | - | - | - | - |
| 41 | trans-.beta.-Ionone | 34.845 | - | - | - | - | - | - | - | - | - | - | - | - | 0.004 | - |
| **E** | **Esters** |  |  |  |  |  |  |  |  |  |  |  |  |  |  |  |
| 42 | 1-Ethylpentyl acetate | 21.388 | - | - | - | - | - | - | - | - | - | 0.003 | - | - | - | - |
| 43 | Butanoic acid, 2-methyl-, 3-methylbutyl ester | 24.786 | - | - | - | - | 0.002 | - | - | - | - | - | - | - | - | - |
| 44 | Butyric acid, 1-propylpentyl ester | 28.973 | 0.162 | 0.008 | 0.008 | 0.022 | 0.007 | 0.037 | 0.076 | - | 0.020 | 0.001 | 1.940 | 0.001 | 0.015 | 0.005 |
| 45 | Hexanoic acid, 3-tridecyl ester | 34.762 | - | - | - | - | - | - | - | - | - | - | - | - | 0.023 | - |
| 46 | Hexanoic acid, 4-octyl ester | 34.752 | 0.139 | - | 0.007 | 0.022 | 0.007 | 0.027 | - | 0.016 | 0.019 | 0.001 | 1.444 | 0.001 | - | - |
| 47 | Hexanoic acid, 6-tridecyl ester | 34.757 | - | - | - | - | - | - | - | - | - | - | - | - | - | 0.002 |
| 48 | Isopropyl palmitate | 46.681 | - | 0.080 | - | - | - | - | - | - | - | - | 0.013 | - | - | - |
| 49 | Pentanoic acid, pentyl ester | 24.548 | - | - | 0.002 | - | - | - | 0.026 | - | 0.011 | - | 0.483 | 0.000 | 0.008 | - |
| 50 | Propanoic acid, propyl ester | 4.888 | - | - | - | - | - | - | - | - | 0.024 | 0.001 | - | 0.001 | - | 0.003 |
| 51 | Valeric acid, 3-tetradecyl ester | 32.075 | - | - | - | - | - | 0.115 | - | - | - | 0.002 | 6.378 | - | - | - |
| 52 | Valeric acid, 4-tridecyl ester | 32.109 | 0.506 | - | - | - | 0.028 | - | - | - | - | - | 0.022 | - | - | - |
| **F** | **Ethers** |  |  |  |  |  |  |  |  |  |  |  |  |  |  |  |
| 53 | n-Amyl ether | 18.973 | - | - | 0.004 | 0.040 | 0.019 | 0.075 | - | 0.028 | 0.057 | 0.002 | 1.456 | 0.003 | 0.035 | 0.022 |
| **G** | **Hydrocarbons** |  |  |  |  |  |  |  |  |  |  |  |  |  |  |  |
| 54 | 1-Propene, 2-methyl- | 1.125 | - | - | - | - | - | - | - | 0.012 | - | - | - | - | - | - |
| 55 | 3-Tridecene, (E)- | 26.826 | - | - | 0.003 | - | - | - | - | - | 0.009 | - | - | - | - | - |
| 56 | 4-Undecene, 4-methyl-, (Z)- | 23.652 | - | - | - | - | - | - | - | - | 0.015 | - | - | - | - | - |
| 57 | 5,11-Diethyl-8-methyl-7,9-dioxapentadecane | 39.348 | - | - | - | - | 0.003 | - | - | 0.014 | - | - | 0.252 | - | - | - |
| 58 | 5-Undecene, 6-methyl- | 22.892 | - | - | - | - | - | 0.024 | - | 0.007 | - | - | 0.398 | 0.001 | - | 0.002 |
| 59 | Cyclohexane, 1,2,3-trimethyl- | 26.685 | - | - | - | - | - | - | - | - | - | - | - | - | - | 0.004 |
| 60 | Cyclohexane, 1,2,3-trimethyl-, (1.alpha.,2.alpha.,3.alpha.)- | 26.685 | - | - | - | - | - | - | - | - | 0.012 | - | - | - | - | - |
| 61 | Cyclohexane, 1,2,3-trimethyl-, (1.alpha.,2.alpha.,3.beta.)- | 26.666 | 0.046 | - | - | - | - | - | - | - | - | 0.000 | - | 0.001 | - | 0.003 |
| 62 | Decane, 3,6-dimethyl- | 48.044 | - | 0.001 | - | - | - | - | - | - | - | - | - | - | - | - |
| 63 | Eicosane | 40.030 | - | 0.001 | - | - | - | 0.003 | - | - | - | - | - | - | - | - |
| 64 | Hexadecane | 37.732 | 0.009 | - | - | - | - | - | - | - | - | - | - | - | - | - |
| 65 | Octadecane | 42.192 | - | 0.001 | - | - | - | - | - | - | - | - | - | - | - | - |
| 66 | Squalene | 53.793 | - | - | - | 0.008 | 0.005 | - | - | - | - | - | 0.153 | - | - | - |
| 67 | Undecane, 6-methyl- | 22.678 | - | 0.007 | - | - | - | 0.040 | - | - | 0.013 | 0.000 | 0.353 | 0.001 | 0.009 | 0.005 |
| **H** | **Phenols** |  |  |  |  |  |  |  |  |  |  |  |  |  |  |  |
| 68 | 2-Methoxy-4-vinylphenol | 29.655 | - | - | - | - | - | - | 0.018 | - | - | - | - | - | - | - |
| 69 | Phenol, 2,4-bis(1,1-dimethylethyl)- | 35.566 | 0.007 | 0.002 | - | - | - | - | - | 0.007 | - | - | 0.080 | 0.000 | 0.002 | - |
| 70 | Scopoletin | 45.327 | - | - | 0.002 | - | - | - | - | - | - | - | - | - | - | - |
| **I** | **Sulfur compounds** |  |  |  |  |  |  |  |  |  |  |  |  |  |  |  |
| 71 | Butane, 1,1'-sulfonylbis- | 33.895 | 0.588 | 0.081 | 0.062 | 0.202 | 0.063 | 0.172 | 0.275 | 0.232 | - | 0.010 | 11.932 | 0.007 | 0.235 | 0.027 |
| 72 | Ethane, 1,1-bis(ethylthio)- | 32.308 | - | - | - | - | - | - | - | - | - | 0.020 | 16.952 | - | - | - |
| 73 | Trisulfide, dipropyl | 30.989 | 0.008 | - | - | - | - | - | - | - | - | - | - | - | - | - |
| **J** | **Nitrogen/Sulfur heterocycles** |  |  |  |  |  |  |  |  |  |  |  |  |  |  |  |
| 74 | 2-Ethyl-4-methylthiazole | 38.301 | - | - | - | - | 0.001 | - | - | - | - | - | - | - | - | - |
| 75 | 4-Methylthiazole | 34.304 | - | - | - | - | 0.003 | - | - | - | - | - | - | - | - | - |
| 76 | Dimethyl palmitamine | 44.354 | - | 0.004 | - | - | - | - | - | - | - | - | - | - | - | - |
| 77 | Thiazole, 2,5-dimethyl- | 40.716 | - | - | - | - | 0.005 | - | - | - | - | - | - | - | - | - |
| 78 | Thiazole, 2-ethyl-4,5-dimethyl- | 19.304 | 0.017 | - | - | - | - | - | - | - | - | - | - | - | 0.013 | 0.007 |
| **K** | **Amides** |  |  |  |  |  |  |  |  |  |  |  |  |  |  |  |
| 79 | 9-Octadecenamide, (Z)- | 52.547 | - | - | 0.004 | - | - | - | - | - | - | - | - | - | - | - |
| **L** | **Others** |  |  |  |  |  |  |  |  |  |  |  |  |  |  |  |
| 80 | 3-Trifluoroacetoxytridecane | 27.084 | - | - | - | - | - | - | - | 0.006 | - | - | - | - | - | - |
| 81 | Tributylamine | 24.786 | - | - | - | - | - | - | - | 0.013 | 0.008 | - | - | - | 0.008 | - |
| 82 | Undecane, 5-methyl- | 37.717 | - | - | - | - | - | - | - | - | - | - | - | - | 0.003 | - |

Note. Values are expressed as ng/g. "-" indicates not detected.

**Supplementary Table S5. Effect of LED pretreatment and ultrasound-assisted convective drying on relative content (%) of volatile compounds of goji berries.**

| **No.** | **Compounds** | **RT (min)** | **Fresh** | **Sun-dried** | **US-Con70** | **US-Con80** | **US-Con90** | **US-BL70** | **US-BL80** | **US-BL90** | **US-YL70** | **US-YL80** | **US-YL90** | **US-RL70** | **US-RL80** | **US-RL90** |
| --- | --- | --- | --- | --- | --- | --- | --- | --- | --- | --- | --- | --- | --- | --- | --- | --- |
| **A** | **Acids** |  |  |  |  |  |  |  |  |  |  |  |  |  |  |  |
| 1 | 6-Octadecenoic acid, (Z)- | 48.711 | - | - | - | - | 0.049 | 0.048 | - | - | - | - | 0.127 | 0.02 | - | - |
| 2 | 9,12-Octadecadienoic acid (Z,Z)- | 48.652 | - | - | 11.23 | 0.143 | 0.039 | 0.037 | 0.111 | 0.219 | - | - | 0.127 | - | - | - |
| 3 | 9-Hexadecenoic acid | 45.035 | - | - | - | - | - | - | - | - | - | - | - | - | - | 0.066 |
| 4 | 9-Octadecenoic acid, (E)- | 48.701 | - | 0.069 | - | 0.237 | - | - | 0.188 | - | - | - | 0.127 | - | - | - |
| 5 | Docosanoic acid | 57.216 | 0.122 | - | - | - | - | - | - | - | - | - | - | - | - | - |
| 6 | Octadec-9-enoic acid | 48.750 | 0.637 | - | - | - | - | - | - | 0.522 | - | - | 0.127 | - | - | - |
| 7 | Octadecanoic acid | 49.163 | 0.243 | - | 2.772 | 0.094 | 0.012 | - | 0.091 | 0.126 | - | - | 0.063 | - | - | - |
| 8 | Oleic Acid | 48.896 | - | - | 3.947 | - | - | - | - | - | - | - | 0.127 | - | - | - |
| 9 | Palmitoleic acid | 45.021 | - | 0.1 | 0.269 | 0.128 | - | - | 0.04 | - | - | - | 0.034 | - | - | - |
| 10 | Pentadecanoic acid | 43.443 | - | 0.072 | - | 0.058 | - | - | 0.024 | - | - | - | 0.032 | - | - | - |
| 11 | Tetradecanoic acid | 41.364 | - | 0.204 | 0.196 | 0.123 | - | - | 0.043 | - | - | - | 0.042 | 0.068 | - | 0.068 |
| 12 | n-Decanoic acid | 31.218 | - | 0.064 | - | - | - | - | - | - | - | - | - | - | - | - |
| 13 | n-Hexadecanoic acid | 45.444 | 1.531 | 0.554 | 4.351 | 0.562 | 0.046 | 0.037 | 0.346 | 0.288 | 0.186 | 0.209 | 0.346 | 0.386 | 0.182 | 0.32 |
| **B** | **Alcohols** |  |  |  |  |  |  |  |  |  |  |  |  |  |  |  |
| 14 | (S)-(+)-5-Methyl-1-heptanol | 18.413 | - | - | - | - | - | - | - | - | - | 4.057 | 6.255 | - | - | 4.962 |
| 15 | 1-Pentanol | 3.593–4.800 | - | 12.377 | 7.488 | 2.592 | 1.458 | 2.406 | 6.231 | 7.987 | 10.72 | 8.061 | 9.109 | 9.681 | 9.081 | 9.723 |
| 16 | 1-Propanol | 1.275–1.441 | - | - | 1.083 | 0.187 | 0.186 | 0.17 | 0.61 | 1.501 | 6.419 | 6.6 | 2.757 | 7.789 | 3.6 | 3.232 |
| 17 | 2,4-Dimethyl-1-hepten-4-ol | 17.026 | - | 0.285 | - | 0.027 | 0.027 | - | - | 0.121 | 0.298 | 0.172 | 0.676 | 0.226 | - | 0.184 |
| 18 | 2-Heptanol, 2-methyl- | 10.843 | - | 2.072 | 1.715 | 0.477 | 0.32 | 0.225 | 0.77 | 2.045 | - | 0.813 | 0.097 | 2.63 | 2.559 | 2.516 |
| 19 | 2-Hexanol, 2,3-dimethyl- | 10.828 | - | - | 0.21 | - | - | 0.052 | - | - | 3.391 | 1.899 | 0.047 | - | - | - |
| 20 | 2-Pentanol, 3-ethyl-2-methyl- | 13.068 | - | - | - | - | - | - | 0.137 | - | - | - | - | - | - | - |
| 21 | 3-Heptanol, 4-methyl- | 3.588 | 2.469 | 1.111 | - | 64.024 | 0.11 | 0.213 | - | 0.94 | 2.658 | 1.582 | 22.272 | 1.576 | 1.529 | 1.276 |
| 22 | 3-Heptanol, 5-methyl- | 3.272 | 2.394 | - | - | - | - | 13.766 | - | - | - | - | 0.424 | - | - | - |
| 23 | 3-Heptanol, 6-methyl- | 4.255 | 58.789 | - | - | - | - | 33.136 | 40.566 | - | - | - | 3.068 | - | - | - |
| 24 | 3-Nonanol | 20.701 | - | - | - | - | - | 0.016 | - | - | - | - | - | - | - | - |
| 25 | 4,5-Dimethyl-3-heptanol | 25.799 | 0.35 | - | - | - | - | - | - | - | - | - | - | - | - | - |
| 26 | 5-Ethyl-3-methylhept-1-en-4-ol | 3.496 | 1.834 | - | - | - | 82.909 | 29.4 | - | - | - | - | 1.244 | - | - | - |
| **C** | **Aldehydes** |  |  |  |  |  |  |  |  |  |  |  |  |  |  |  |
| 27 | 2-Ethyl-trans-2-butenal | 5.804 | - | 1.714 | 0.634 | - | 0.148 | - | - | - | - | - | 1.063 | - | - | - |
| 28 | 2-Pentenal, 2-methyl- | 5.711 | - | - | - | 0.199 | - | 0.188 | 0.469 | 0.972 | 1.345 | 1.06 | 1.063 | 1.128 | 1.368 | 1.311 |
| 29 | 4,9,13,17-Tetramethyl-4,8,12,16-octadecatetraenal | 43.02 | - | 0.064 | - | - | - | - | - | - | - | - | - | - | - | - |
| **D** | **Ketones** |  |  |  |  |  |  |  |  |  |  |  |  |  |  |  |
| 30 | 2(4H)-Benzofuranone, 5,6,7,7a-tetrahydro-4,4,7a-trimethyl- | 35.907 | - | 0.074 | - | - | - | - | - | - | - | - | - | - | - | 0.037 |
| 31 | 2-Cyclopenten-1-one, 2,3,4,5-tetramethyl- | 21.169 | - | 0.031 | - | - | - | - | - | - | - | - | - | - | 0.043 | - |
| 32 | 3-Nonanone | 19.431 | - | 0.245 | - | - | - | - | - | - | 0.163 | - | - | 0.196 | - | 0.204 |
| 33 | 3-Octanone | 14.134 | - | 74.91 | 58.919 | 28.961 | 13.286 | 19.257 | 48.909 | 76.138 | 71.869 | 58.708 | 17.892 | 70.963 | 70.318 | 71.982 |
| 34 | 3-Penten-2-one, 4-methyl- | 6.227 | - | - | - | - | - | - | - | - | - | 0.163 | - | 0.16 | - | 0.254 |
| 35 | 4-Nonene, 5-butyl- | 26.709 | - | - | 0.169 | 0.058 | - | - | 0.088 | - | - | 0.09 | 0.297 | - | 0.406 | - |
| 36 | 4H-Pyran-4-one, 2,3-dihydro-3,5-dihydroxy-6-methyl- | 22.001 | - | 0.141 | - | 0.259 | 0.119 | 0.038 | 0.353 | - | 0.196 | 0.425 | 1.625 | - | 1.016 | 0.61 |
| 37 | 5-Decanone | 23.628 | - | - | - | - | - | - | - | - | - | 0.173 | - | - | 0.292 | - |
| 38 | Ar-tumerone | 39.3 | - | - | - | - | - | 0.067 | - | - | - | - | - | - | - | - |
| 39 | Curlone | 40.122 | - | - | - | - | - | 0.032 | - | - | - | - | - | - | - | - |
| 40 | Tumerone | 39.382 | - | - | - | - | - | 0.034 | - | - | - | - | - | - | - | - |
| 41 | trans-.beta.-Ionone | 34.845 | - | - | - | - | - | - | - | - | - | - | - | - | 0.11 | - |
| **E** | **Esters** |  |  |  |  |  |  |  |  |  |  |  |  |  |  |  |
| 42 | 1-Ethylpentyl acetate | 21.388 | - | - | - | - | - | - | - | - | - | 1.158 | - | - | - | - |
| 43 | Butanoic acid, 2-methyl-, 3-methylbutyl ester | 24.786 | - | - | - | - | 0.016 | - | - | - | - | - | - | - | - | - |
| 44 | Butyric acid, 1-propylpentyl ester | 28.973 | 3.451 | 0.247 | 0.583 | 0.138 | 0.064 | 0.067 | 0.196 | - | 0.296 | 0.216 | 1.435 | 0.267 | 0.411 | 0.202 |
| 45 | Hexanoic acid, 3-tridecyl ester | 34.762 | - | - | - | - | - | - | - | - | - | - | - | - | 0.618 | - |
| 46 | Hexanoic acid, 4-octyl ester | 34.752 | 2.968 | - | 0.515 | 0.138 | 0.064 | 0.048 | - | 0.447 | 0.28 | 0.3 | 1.068 | 0.225 | - | - |
| 47 | Hexanoic acid, 6-tridecyl ester | 34.757 | - | - | - | - | - | - | - | - | - | - | - | - | - | 0.095 |
| 48 | Isopropyl palmitate | 46.681 | - | 2.557 | - | - | - | - | - | - | - | - | 0.01 | - | - | - |
| 49 | Pentanoic acid, pentyl ester | 24.548 | - | - | 0.186 | - | - | - | 0.068 | - | 0.157 | - | 0.358 | 0.143 | 0.21 | - |
| 50 | Propanoic acid, propyl ester | 4.888 | - | - | - | - | - | - | - | - | 0.347 | 0.295 | - | 0.444 | - | 0.138 |
| 51 | Valeric acid, 3-tetradecyl ester | 32.075 | - | - | - | - | - | 0.205 | - | - | - | 0.931 | 4.718 | - | - | - |
| 52 | Valeric acid, 4-tridecyl ester | 32.109 | 10.793 | - | - | - | 0.251 | - | - | - | - | - | 0.017 | - | - | - |
| **F** | **Ethers** |  |  |  |  |  |  |  |  |  |  |  |  |  |  |  |
| 53 | n-Amyl ether | 18.973 | - | - | 0.28 | 0.255 | 0.173 | 0.133 | - | 0.757 | 0.837 | 0.732 | 1.077 | 0.851 | 0.944 | 0.894 |
| **G** | **Hydrocarbons** |  |  |  |  |  |  |  |  |  |  |  |  |  |  |  |
| 54 | 1-Propene, 2-methyl- | 1.125 | - | - | - | - | - | - | - | 0.328 | - | - | - | - | - | - |
| 55 | 3-Tridecene, (E)- | 26.826 | - | - | 0.233 | - | - | - | - | - | 0.131 | - | - | - | - | - |
| 56 | 4-Undecene, 4-methyl-, (Z)- | 23.652 | - | - | - | - | - | - | - | - | 0.223 | - | - | - | - | - |
| 57 | 5,11-Diethyl-8-methyl-7,9-dioxapentadecane | 39.348 | - | - | - | - | 0.026 | - | - | 0.388 | - | - | 0.186 | - | - | - |
| 58 | 5-Undecene, 6-methyl- | 22.892 | - | - | - | - | - | 0.042 | - | 0.185 | - | - | 0.295 | 0.32 | - | 0.088 |
| 59 | Cyclohexane, 1,2,3-trimethyl- | 26.685 | - | - | - | - | - | - | - | - | - | - | - | - | - | 0.169 |
| 60 | Cyclohexane, 1,2,3-trimethyl-, (1.alpha.,2.alpha.,3.alpha.)- | 26.685 | - | - | - | - | - | - | - | - | 0.181 | - | - | - | - | - |
| 61 | Cyclohexane, 1,2,3-trimethyl-, (1.alpha.,2.alpha.,3.beta.)- | 26.666 | 0.988 | - | - | - | - | - | - | - | - | 0.141 | - | 0.312 | - | 0.117 |
| 62 | Decane, 3,6-dimethyl- | 48.044 | - | 0.046 | - | - | - | - | - | - | - | - | - | - | - | - |
| 63 | Eicosane | 40.030 | - | 0.04 | - | - | - | 0.005 | - | - | - | - | - | - | - | - |
| 64 | Hexadecane | 37.732 | 0.197 | - | - | - | - | - | - | - | - | - | - | - | - | - |
| 65 | Octadecane | 42.192 | - | 0.032 | - | - | - | - | - | - | - | - | - | - | - | - |
| 66 | Squalene | 53.793 | - | - | - | 0.049 | 0.043 | - | - | - | - | - | 0.113 | - | - | - |
| 67 | Undecane, 6-methyl- | 22.678 | - | 0.237 | - | - | - | 0.071 | - | - | 0.183 | 0.15 | 0.261 | 0.201 | 0.241 | 0.198 |
| **H** | **Phenols** |  |  |  |  |  |  |  |  |  |  |  |  |  |  |  |
| 68 | 2-Methoxy-4-vinylphenol | 29.655 | - | - | - | - | - | - | 0.048 | - | - | - | - | - | - | - |
| 69 | Phenol, 2,4-bis(1,1-dimethylethyl)- | 35.566 | 0.152 | 0.059 | - | - | - | - | - | 0.192 | - | - | 0.059 | 0.055 | 0.048 | - |
| 70 | Scopoletin | 45.327 | - | - | 0.144 | - | - | - | - | - | - | - | - | - | - | - |
| **I** | **Sulfur compounds** |  |  |  |  |  |  |  |  |  |  |  |  |  |  |  |
| 71 | Butane, 1,1'-sulfonylbis- | 33.895 | 12.537 | 2.572 | 4.799 | 1.291 | 0.57 | 0.307 | 0.714 | 6.329 | - | 4.111 | 8.826 | 2.362 | 6.379 | 1.086 |
| 72 | Ethane, 1,1-bis(ethylthio)- | 32.308 | - | - | - | - | - | - | - | - | - | 7.956 | 12.539 | - | - | - |
| 73 | Trisulfide, dipropyl | 30.989 | 0.178 | - | - | - | - | - | - | - | - | - | - | - | - | - |
| **J** | **Nitrogen/Sulfur heterocycles** |  |  |  |  |  |  |  |  |  |  |  |  |  |  |  |
| 74 | 2-Ethyl-4-methylthiazole | 38.301 | - | - | - | - | 0.012 | - | - | - | - | - | - | - | - | - |
| 75 | 4-Methylthiazole | 34.304 | - | - | - | - | 0.029 | - | - | - | - | - | - | - | - | - |
| 76 | Dimethyl palmitamine | 44.354 | - | 0.121 | - | - | - | - | - | - | - | - | - | - | - | - |
| 77 | Thiazole, 2,5-dimethyl- | 40.716 | - | - | - | - | 0.042 | - | - | - | - | - | - | - | - | - |
| 78 | Thiazole, 2-ethyl-4,5-dimethyl- | 19.304 | 0.367 | - | - | - | - | - | - | - | - | - | - | - | 0.361 | 0.267 |
| **K** | **Amides** |  |  |  |  |  |  |  |  |  |  |  |  |  |  |  |
| 79 | 9-Octadecenamide, (Z)- | 52.547 | - | - | 0.276 | - | - | - | - | - | - | - | - | - | - | - |
| **L** | **Others** |  |  |  |  |  |  |  |  |  |  |  |  |  |  |  |
| 80 | 3-Trifluoroacetoxytridecane | 27.084 | - | - | - | - | - | - | - | 0.159 | - | - | - | - | - | - |
| 81 | Tributylamine | 24.786 | - | - | - | - | - | - | - | 0.357 | 0.12 | - | - | - | 0.21 | - |
| 82 | Undecane, 5-methyl- | 37.717 | - | - | - | - | - | - | - | - | - | - | - | - | 0.075 | - |

Note. Values are expressed as relative content (%). "-" indicates not detected.

**References Table 1**

[1]	H. Song, J. Bi, Q. Chen, M. Zhou, X. Wu, J. Song, Structural and health functionality of dried goji berries as affected by coupled dewaxing pretreatment and hybrid drying methods, Int. J. Food Prop. 21 (2018) 2527–2538. <https://doi.org/10.1080/10942912.2018.1536148>.

[2]	Q. Zhang, F. Wan, Z. Zang, C. Jiang, Y. Xu, X. Huang, Effect of ultrasonic far-infrared synergistic drying on the characteristics and qualities of wolfberry (Lycium barbarum L.), Ultrason. Sonochem. 89 (2022). <https://doi.org/10.1016/j.ultsonch.2022.106134>.

[3]	B. Turan, Z.H. Tekin-Cakmak, S. Kayacan Çakmakoglu, S. Karasu, M.Z. Kasapoglu, E. Avci, Effect of Different Drying Techniques on Total Bioactive Compounds and Individual Phenolic Composition in Goji Berries, Processes 11 (2023). https://doi.org/10.3390/pr11030754.

[4]	A.A. Zhang, L. Xie, Q.H. Wang, M.Q. Xu, Y. Pan, Z.A. Zheng, W.Q. Lv, H.W. Xiao, Effect of the ripening stage on the pulsed vacuum drying behavior of goji berry (Lycium barbarum L.): Ultrastructure, drying characteristics, and browning mechanism, Food Chem. 442 (2024). https://doi.org/10.1016/j.foodchem.2024.138489.

**References Table 2**

[5]	A. El-Beltagy, G.R. Gamea, A.H.A. Essa, Solar drying characteristics of strawberry, J. Food Eng. 78 (2007) 456–464. https://doi.org/10.1016/j.jfoodeng.2005.10.015.

[6]	E.O.M. Akoy, Experimental characterization and modeling of thin-layer drying of mango slices, Int. Food Res. J. 21 (2014) 1911–1917. https://www.researchgate.net/profile/Elamin-Akoy/publication/273061769_Experimental_characterization_and_modeling_of_thin-layer_drying_of_mango_slices/links/54f6195a0cf27d8ed71d5c74/Experimental-characterization-and-modeling-of-thin-layer-drying-of-mango-slices.pdf (accessed June 19, 2026).

[7]	N. Hashim, O. Daniel, E. Rahaman, A Preliminary Study: Kinetic Model of Drying Process of Pumpkins (Cucurbita Moschata) in a Convective Hot Air Dryer, Agric. Agric. Sci. Procedia 2 (2014) 345–352. https://doi.org/10.1016/j.aaspro.2014.11.048.

[8]	M.S. Zenoozian, H. Feng, S.M.A. Razavi, F. Shahidi, H.R. Pourreza, Image analysis and dynamic modeling of thin-layer drying of osmotically dehydrated pumpkin, J. Food Process. Preserv. 32 (2008) 88–102. https://doi.org/10.1111/j.1745-4549.2007.00167.x.

[9]	K. Kulwinder, S. A.K., Drying kinetics and quality characteristics of beetroot slices under hot air followed by microwave finish drying, African J. Agric. Res. 9 (2014) 1036–1044. https://doi.org/10.5897/AJAR2013.

[10]	M. Campus, A.- Bellevue, Mathematical modeling of thin layer drying kinetics of apples slices, Int. Food Res. J. 19 (2006) 1949–1958. https://doi.org/10.1051/IUFoST.

[11]	K. Sacilik, Effect of drying methods on thin-layer drying characteristics of hull-less seed pumpkin (Cucurbita pepo L.), J. Food Eng. 79 (2007) 23–30. https://doi.org/10.1016/j.jfoodeng.2006.01.023.

[12]	C.L. Hii, C.L. Law, M. Cloke, Modeling using a new thin layer drying model and product quality of cocoa, J. Food Eng. 90 (2009) 191–198. https://doi.org/10.1016/j.jfoodeng.2008.06.022.

[13]	K. An, D. Zhao, Z. Wang, J. Wu, Y. Xu, G. Xiao, Comparison of different drying methods on Chinese ginger (Zingiber officinale Roscoe): Changes in volatiles, chemical profile, antioxidant properties, and microstructure, Food Chem. 77 (2016) 4716–4724. https://doi.org/10.1016/j.phytochem.2015.07.012.

[14]	P.L. Gan, P.E. Poh, Investigation on the Effect of Shapes on the Drying Kinetics and Sensory Evaluation Study of Dried Jackfruit, Int. J. Sci. Eng. 7 (2014) 193–198. https://doi.org/10.12777/ijse.7.2.193-198.

[15]	İ. Doymaz, S. Karasu, M. Baslar, Effects of infrared heating on drying kinetics, antioxidant activity, phenolic content, and color of jujube fruit, J. Food Meas. Charact. 10 (2016) 283–291. https://doi.org/10.1007/s11694-016-9305-4.

[16]	A.O. Omolola, A.I.O. Jideani, P.F. Kapila, Modeling microwave drying kinetics and moisture diffusivity of mabonde banana variety, Int. J. Agric. Biol. Eng. 7 (2014) 107–113. https://doi.org/10.3965/j.ijabe.20140706.013.

[17]	W.P. da Silva, C.M.D.P.S. e Silva, F.J.A. Gama, J.P. Gomes, Mathematical models to describe thin-layer drying and to determine drying rate of whole bananas, J. Saudi Soc. Agric. Sci. 13 (2014) 67–74. https://doi.org/10.1016/j.jssas.2013.01.003.
